# Accelerated disease progression and robust innate host response in aged SIVmac239-infected Chinese rhesus macaques is associated with enhanced immunosenescence

**DOI:** 10.1038/s41598-017-00084-0

**Published:** 2017-02-24

**Authors:** Hong-Yi Zheng, Ming-Xu Zhang, Min Chen, Jin Jiang, Jia-Hao Song, Xiao-Dong Lian, Ren-Rong Tian, Xiao-Liang Zhang, Lin-Tao Zhang, Wei Pang, Gao-Hong Zhang, Yong-Tang Zheng

**Affiliations:** 1 Key Laboratory of Animal Models and Human Disease Mechanisms of Chinese Academy of Sciences &Yunnan Province, Kunming Institute of Zoology, Chinese Academy of Sciences, Kunming, Yunnan 650223 China; 2 School of Life Sciences, University of Science and Technology of China, Hefei, Anhui 230026 China; 3 Kunming College of Life Science, University of Chinese Academy of Sciences, Kunming, Yunnan 650204 China; 4 Institute of Health Sciences, Anhui University, Hefei, Anhui 230601 China; 5 Kunming Primate Research Center, Kunming Institute of Zoology, Chinese Academy of Sciences, Kunming, Yunnan 650223 China

## Abstract

The elderly population infected with HIV-1 is often characterized by the rapid AIDS progression and poor treatment outcome, possibly because of immunosenescence resulting from both HIV infection and aging. However, this hypothesis remains to be fully tested. Here, we studied 6 young and 12 old Chinese rhesus macaques (ChRM) over the course of three months after simian immunodeficiency virus (SIV) SIVmac239 infection. Old ChRM showed a higher risk of accelerated AIDS development than did young macaques, owing to rapidly elevated plasma viral loads and decreased levels of CD4^+^ T cells. The low frequency of naïve CD4^+^ T cells before infection was strongly predictive of an increased disease progression, whereas the severe depletion of CD4^+^ T cells and the rapid proliferation of naïve lymphocytes accelerated the exhaustion of naïve lymphocytes in old ChRM. Moreover, in old ChRM, a robust innate host response with defective regulation was associated with a compensation for naïve T cell depletion and a high level of immune activation. Therefore, we suggest that immunosenescence plays an important role in the accelerated AIDS progression in elderly individuals and that SIV-infected old ChRM may be a favorable model for studying AIDS pathogenesis and researching therapies for elderly AIDS patients.

## Introduction

HIV is increasingly and extensively spreading among older individuals. Because an increased risk of HIV infection has been found in the elderly population, HIV-1-infected elderly patients have drawn great attention recently^1^. In 2015, more than half of all HIV-1-infected individuals in the USA were over 50 years of age, and it has been predicted that this percentage will increase to 70% by 2020^2^. Despite successful viral suppression by ART, elderly HIV+ patients still have a slower rate of immune reconstitution and a higher mortality 4 years after seroconversion than do younger patients^3^.

Increasing evidence suggests that a variety of HIV infection-induced immunologic alterations are similar to those seen in uninfected elderly people. Most of these alterations often exhibit age-related abnormalities in adaptive immunity, including an accelerated decrease in T-cell renewal and an increase in B-cell exhaustion, an accumulation of terminally differentiated memory cells with poor proliferative responses, a shortened replication history, constricted diversity within the T-cell receptor (TCR) and B-cell receptor (BCR) repertoire and decreased responsiveness to vaccines. These outcomes ultimately result in a general decline in immunity, thus gradually giving rise to immunosenescence^4–6^.

Immunosenescence is often characterized by thymic involution and reduced output and function of hematopoietic stem cells (HSCs), thus resulting in a poor response to vaccination and an increased susceptibility to infection in the elderly^7, 8^. According to the Swedish OCTO and NONA immune longitudinal studies, immune risk profiles (IRP) associated with low-grade inflammation, including an inverse ratio of CD4/CD8, a reduction in B cells, the depletion of naïve T cells, an increased frequency of well^−^differentiated CD28^−^CD57^+^ T cells and the expansion of CMV-specific oligoclonal cells, have strong associations with morbidity and mortality in the elderly^9, 10^. Several studies have demonstrated that classical markers of immunosenescence such as a high frequency CD28^−^ T cells and CD31^−^ naïve T cells strongly predict subsequent disease progression, less CD4^+^ T cell restoration after 2 years of treatment and poor responsiveness to vaccination after ART independent of other predictors in HIV-1+ patients^11–13^. Thus, immunosenescence due to both HIV-1 infection and aging may explain the accelerated development of AIDS and poor outcomes in older HIV-1-infected patients.

Most of the findings described above lack evidence from longitudinal studies conducted throughout the entire course of HIV-1 infection. Fortunately, Asian macaques such as rhesus macaques (RM) and pigtailed macaques (PTM) infected with multiple SIV strains or related artificial chimeras (HSIV/SHIV) are desirable animal models to study the pathogenesis of HIV/AIDS as well as its prevention and treatment^14^. Furthermore, existing evidence indicates that aged RM may serve as a translational model for immunosenescence research, because their immune system ages in a manner that is similar to the process in humans^15–17^. Recent studies have detected a high level of viral replication in aged RM with severe acute respiratory syndromes (SARS) or influenza due to increased inflamm-aging^18, 19^. Surprisingly, some AIDS cases have even been found in SIV-infected natural hosts that are generally regarded as non-pathogenic models and that are primarily aged animals^20^. It has been suggested that aged non-human primates have a high susceptibility to various types of infections. Our previous studies have revealed that ChRM act either as an excellent model for HIV-1 infection in humans or as a favorable model to research the mechanisms of immunosenescence^21–23^. Thus, there are reasons for believing that old ChRM infected with SIV might be a favorable model to study the pathogenesis and treatment of the HIV-1-infected elderly population. In this study, we compared the immunological, virological and gene-expression dynamics between young and old ChRM during early SIVmac239 infection to identify the characteristics of elderly AIDS patients and to explore the interactions between immunosenescence and HIV/SIV infection.

## Results

### SIVmac239 infection induces a higher risk of rapid disease progression in old compared with young ChRM

**Figure 1:**
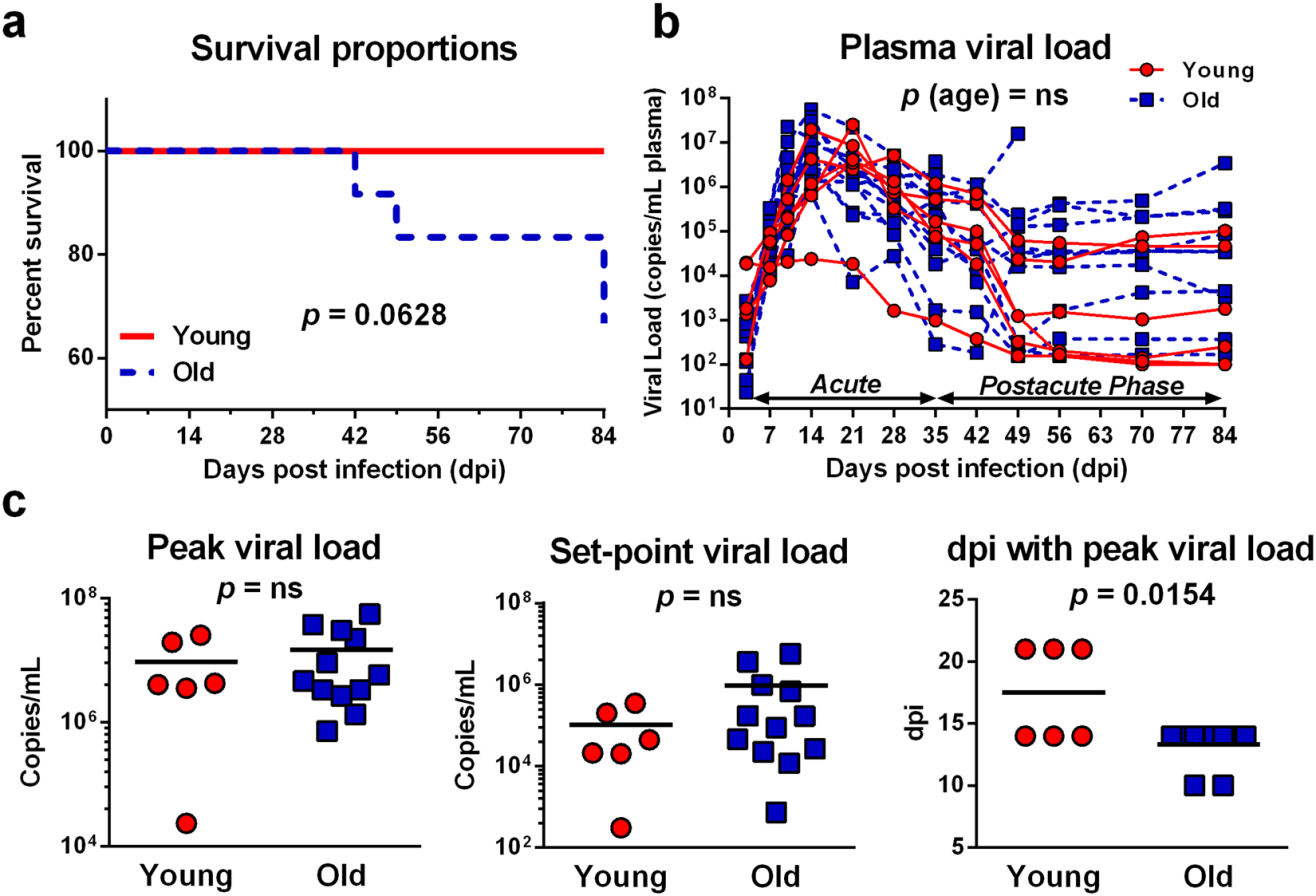
Disease status of young and old ChRM during early SIV infection. (**a**) Kaplan–Meier survival curve comparing SIVmac239-infected young macaques (n = 6, red solid line) with old macaques (n = 12, blue dashed line) during the 84 days after infection. The *P* value is given by the log rank (Mantel–Cox) test. (**b**) Plasma viral loads are expressed as SIV RNA copies/ml and are shown for every animal in the young (red circle) and old (blue square) groups. The data are shown as the mean ± SEM. The comparison between the two groups was performed by using two-way ANOVA. (**c**) Peak and set-point levels of viral load and days with peak viral load were compared between the young and old macaques. *P* values were determined by using the Mann-Whitney t-test.

To explore the pathogenesis of acute HIV infection in elderly patients, we compared 6 young and 12 old Chinese rhesus macaques infected with SIVmac239 in the first 3 months after infection. Thirty-five days post-infection (dpi), 4 old macaques without the Mamu-B*03, Mamu-B*08 and Mamu-B*17 alleles gradually died of severe diarrhea and wasting disease even in spite of antibiotic treatment, whereas 4 young macaques lacking the protective MHC class I alleles remained alive during early infection (Fig. 1a and Supplementary Table S1). To determine whether this increased risk of death was associated with a high level of viremia, we monitored the plasma viral loads of all of the animals. Similarly to the 9.63 × 10^6^ copies/ml plasma in the young animals, a mean peak viral load of 1.58 × 10^7^ copies/ml plasma was found in the acute phase in the old ChRM (*P* = 0.6468). Throughout the infection, no significant differences in viral load were detected between the young and old groups (Fig. 1b). However, the old macaques reached their peak earlier, at 10 or 14 dpi, compared with the delayed peak at 14 or 21 dpi in the young macaques (*P* = 0.0154) (Fig. 1c). Furthermore, in contrast to the survivors, the macaques that died displayed a more robust viral replication as indicated by higher viral loads from 35 dpi to death (Supplementary Fig. S2). Because chronic diarrhea induced by increased intestinal permeability occurred during the progression of SIV infection^24^, it is conceivable that old ChRM may have a higher risk of rapid progression of AIDS.

### CD4^+^ T cells are rapidly depleted in the peripheral blood of old ChRM after SIV infection

**Figure 2:**
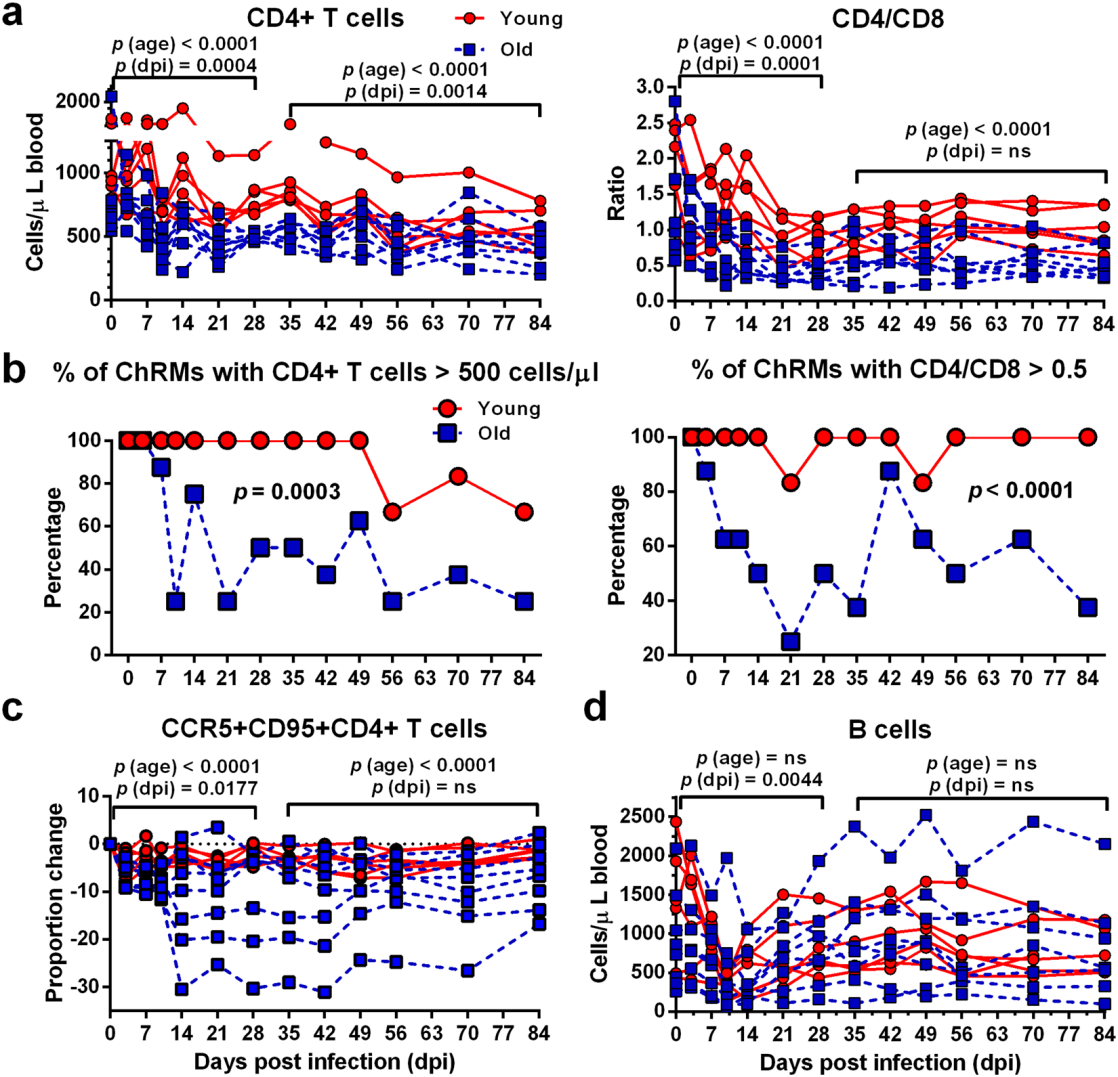
Old ChRM display an increased depletion of CD4^+^ T cells, as compared with young ChRM. (**a**) Longitudinal assessment of CD4^+^ T cell numbers and the CD4/CD8 ratio in the peripheral blood of the young (n = 6, red circle) and old (n = 8, blue square) groups during the 84 days after infection (dpi) is shown in dot plots. (**b**) The frequency of animals with CD4^+^ T cells > 500 cells/μl blood or a CD4/CD8 ratio > 0.5 within the young and old groups. *P* values were determined using the Mann-Whitney t-test. (**c**) The dynamics of CCR5 expression by CD95^+^CD4^+^ memory T cells in the peripheral blood is shown as the differences in frequency at each dpi relative to day 0. The data are shown as the mean ± SEM. (**d**) Longitudinal assessment of B-cell levels in the peripheral blood of ChRM during early SIV infection. *P* (dpi) < 0.05, statistically significant differences over the acute or postacute phase, as determined by two-way ANOVA. *P* (age) < 0.05, statistically significant differences between the young and old groups, as determined by two-way ANOVA.

In order to accurately evaluate the differences between young and old ChRM during early SIV infection, we only analyzed the data from 6 young and 8 old animals that were still alive after 84 dpi. Before infection, old ChRM had a significantly lower number of CD4^+^ T cells (869.9 cells/μl) and a higher frequency of CCR5^+^CD95^+^CD4^+^ memory T cells (18.60%) than did young macaques (1069.0 cells/μl and 10.09%, respectively; *P* = 0.02 and 0.008, respectively). SIVmac239 infection induced a more dramatic decrease in the CD4^+^ T cell count (265.6 CD4^+^ T cells/μl) and CD4/CD8 ratio (0.36) in old ChRM compared with young macaques (468.1 cells/μl and 0.74; *P* = 0.0027 and 0.02, respectively) (Fig. 2a).

A CD4^+^ T cell count less than 500 cells/μl and a severe inversion of CD4/CD8 (<0.5) in the peripheral blood are believed to be associated with an increased risk of AIDS progression^25, 26^. We found that a higher frequency of old macaques were within these ranges, as compared with young animals after infection (Fig. 2b). Moreover, compared with the young macaques, the old ChRM had a more pronounced and a more proportional reduction of CCR5^+^CD4^+^ memory T cells relative to the pre-infection level. This result is further supported by a previous finding that SIVmac239 preferentially infects CCR5^+^CD4^+^ memory T cells and induces the rapid depletion of CD4^+^ central memory T cells^27^ (Fig. 2c). B cells were depleted quickly and to a great extent both in young and old macaques during only the acute phase (Fig. 2d). No statistically significant changes were detected in CD8^+^ T cells over time, although the old ChRM had more CD8^+^ T cells than did the young macaques (Supplementary Fig. S3).

### SIV infection induces the rapid proliferation of naïve T cells in old ChRM

**Figure 3:**
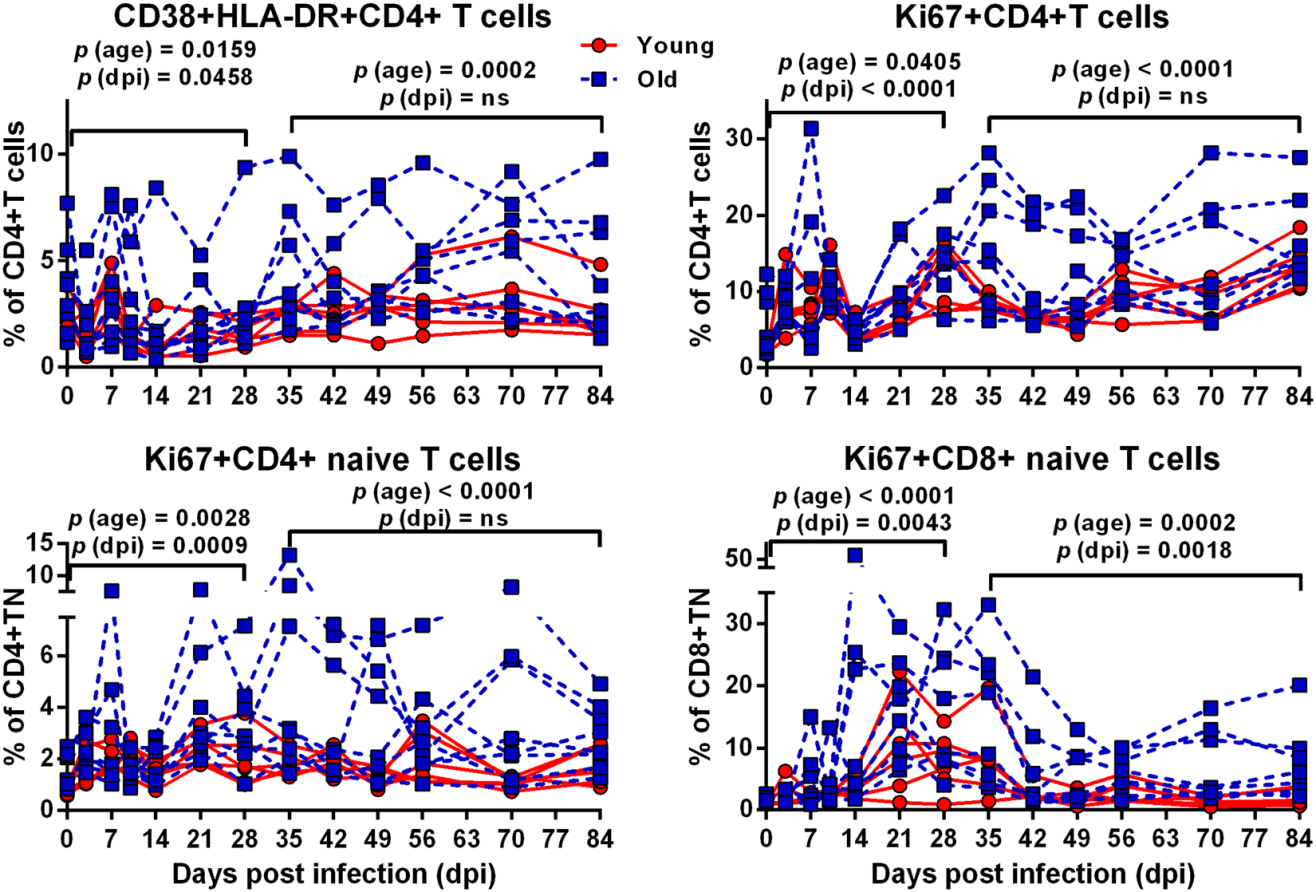
The naïve T cell subsets of old ChRM have an increased level of proliferation. (**a**) Dot plots shows increased levels of CD38^+^HLA-DR^+^CD4^+^ T cells, Ki67^+^CD4^+^ T cells, Ki67^+^CD4^+^ naïve T cells, Ki67^+^CD8^+^ T cells and Ki67^+^CD8^+^ naïve T cells in old (n = 8, blue square) compared with young (n = 6, red circle) animals during early infection. *P* (dpi) < 0.05, statistically significant differences over the acute or postacute phase, as determined by two-way ANOVA. *P* (age) < 0.05, statistically significant differences between the young and old groups, as determined by two-way ANOVA.

**Figure 4:**
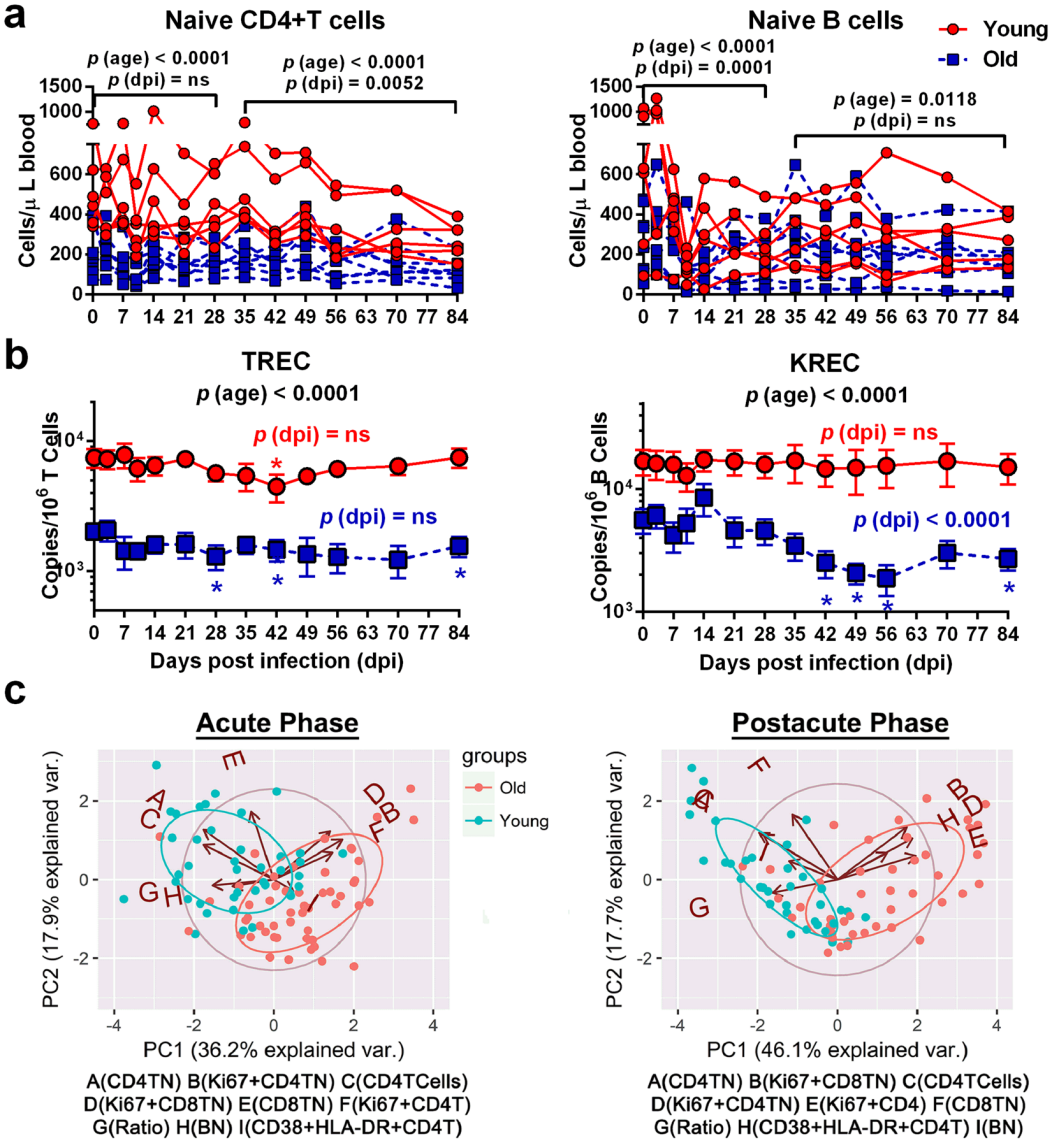
Old ChRM display a decreased number of naïve CD4^+^ T cells and B cells, as compared with young macaques. (**a**) The absolute number of naïve CD4^+^ T and B cells in the peripheral blood of young (n = 6, red circle) and old macaques (n = 8, blue square) is shown in scatter plots. (**b**) Comparison of the TREC (T cell receptor rearrangement excision circle) content in T cells or the KREC (kappa-deletion recombination excision circle) content in B cells from PBMCs in young and old macaques. The data are shown as the mean ± SEM. *p* (dpi) < 0.05 with color, statistically significant differences over the entire infection time, as determined by one-way ANOVA in the young (red) or old (blue) group. *P* (age) < 0.05, statistically significant differences between the young and old groups, as determined by two-way ANOVA. (**c**) Principal component analysis (PCA) of an immunophenotype data set in the acute and postacute phase was calculated by a varimax rotation method in package psych and graphed by package ggbiplot2 with R. Each dot represents a sample from a time point that is plotted against its expression levels for 9 variables that differed over time and between age groups, as determined by two-way ANOVA. The variables are shown in capital letters and are arranged alphabetically according to their communalities from high to low. CD4TCells, CD4^+^ T cell number; CD4TN, naïve CD4^+^ T cell number; CD8TN, naïve CD8^+^ T cell number; Ratio, CD4/CD8; BN, naïve B cell number; Ki67^+^CD4TN, % of Ki67^+^CD4^+^ naïve T cells; Ki67^+^CD8TN, % of Ki67^+^CD8^+^ naïve T cells; Ki67^+^CD4T, % of Ki67^+^CD4^+^ T cells; CD38^+^HLA-DR^+^CD4T, % of CD38^+^HLA-DR^+^CD4^+^ T cells.

Our data reveal that SIVmac239 profoundly induces elevated levels of immune activation markers in most T-cell and B-cell subsets during early infection. Although the levels of these parameters did not display significant differences between the age groups prior to infection, there was a more dramatic increase in the frequency of CD38^+^HLA-DR^+^CD4^+^, Ki67^+^CD4^+^, Ki67^+^CD4^+^ naïve and Ki67^+^CD8^+^ naïve T cells in the old ChRM than in the young macaques after infection (Supplementary Tables S2 and S3). These activated subsets were rapidly up-regulated in the acute phase and tended to be constant in developing into the post-acute phase; however, they were still more highly expressed in old macaques over time (Fig. 3). A principal component analysis (PCA) indicated that the Ki67 expression in naïve T cells was responsible for a large proportion of the differences between the young and old macaques during both the acute and post-acute phases, whereas the difference in CD38^+^HLA-DR^+^CD4^+^ T cells had a negligible effect on these differences (Fig. 4c).

### A rapid disease in progression in old ChRM is associated with a decreased number of naïve lymphocytes

After the frequency of naïve CD4^+^ T cells had fallen to its lowest point 10 dpi, it quickly reached a higher and more steady level than the pre-infection level. This increase in frequency was not due to the increased number of naïve CD4^+^ T cells; instead, it was most probably due to a significant reduction in the central memory (CM) subset. In contrast, SIV infection induced a decreased frequency of the naïve subset and was accompanied by a significant increase in the switched memory (SM) subset in B cells after 10 dpi (Supplementary Fig. S2). Furthermore, we found a lower number of naïve CD4^+^ T cells and naïve B cells than we found the other subsets in old ChRM during infection (Supplementary Tables S2 and S3).

In agreement with the CD4^+^ T cell numbers, the old ChRM also showed a rapid decrease in naïve CD4^+^ T and B cells during acute infection. Before infection, old ChRM had a significantly lower number of naïve CD4^+^ T cells (213.8 cells/μl) than did young macaques (503.4 cells/μl, *P* = 0.008). SIVmac239 infection induced a more dramatic decrease in the naïve CD4^+^ T cell count (67.0 cells/μl) in old ChRM compared with young macaques (202.8 cells/μl, *P* = 0.0009). However, no statistically significant differences of naïve B cell count were detected in pre-infection and nadir levels between young and old macaques. Moreover, according to a PCA, the naïve CD4^+^ T cell numbers accounted for the majority of variances between the young and the old macaques in both the acute and post-acute phases (Fig. 4c).

To determine whether the decrease in naïve lymphocytes was due to the SIV-induced dysfunction of thymic and myeloid output, we measured the concentrations of TREC or KREC in T cells or B cells, respectively, as previously described^28^. Old ChRM displayed an impairment in thymic and myeloid output throughout early infection. Moreover, although it had no significant effects on TREC and KREC in young ChRM, SIV infection nonetheless induced a dramatic decrease in KREC concentration from 5588 copies/10^6^ B cells before infection to 2694 copies/10^6^ B cells at 84 dpi (*P* = 0.0307) and a decrease in TREC concentration from 2005 copies/10^6^ T cells before infection to 1552 copies/10^6^ T cells at 84 dpi (*P* = 0.0017) in old macaques (Fig. 4b).

### Increased immunosenescence in old ChRM predicts further disease progression

**Figure 5:**
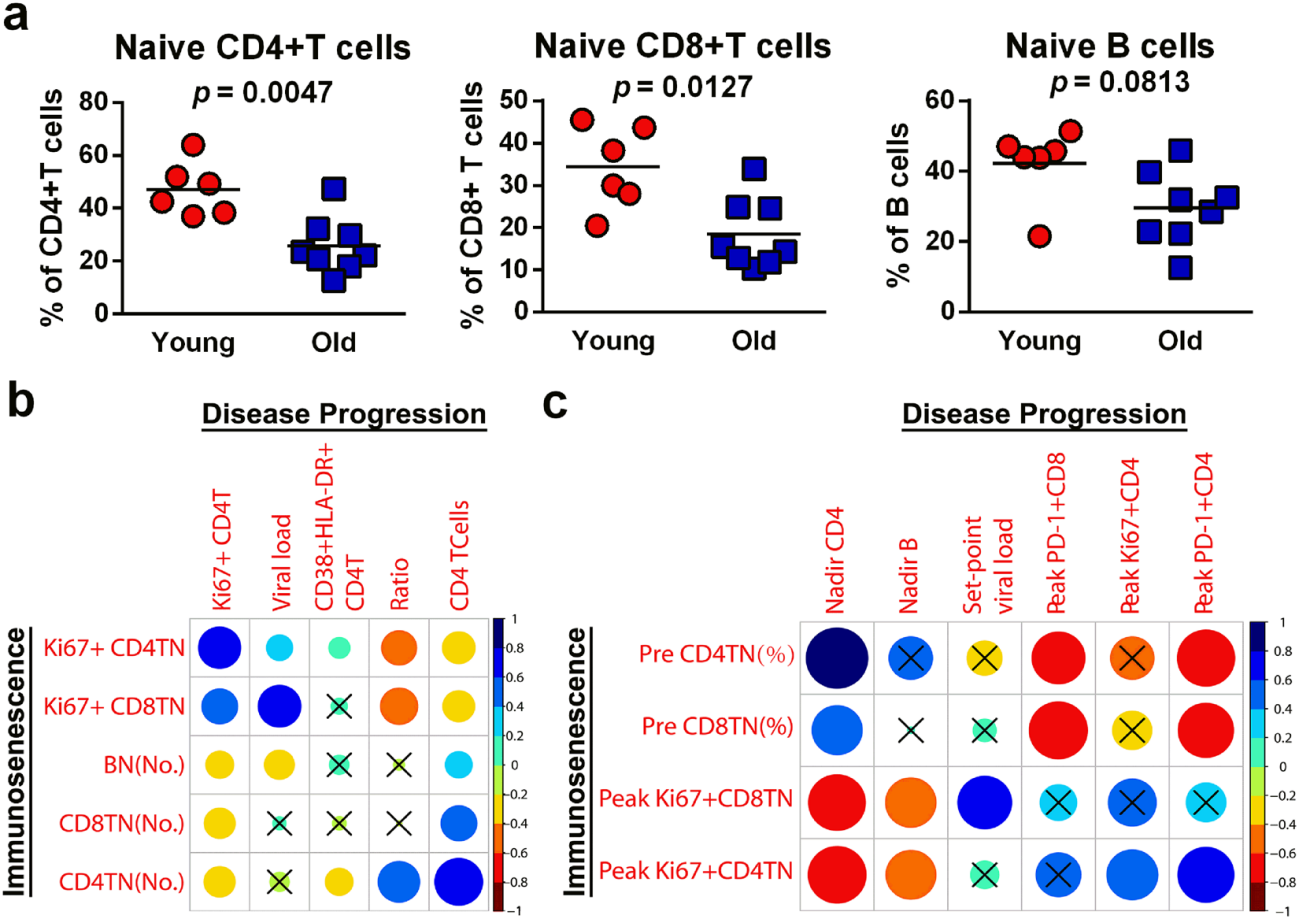
Immunosenescence predicts accelerated disease progression in old ChRM. (**a**) Old ChRM have a significantly lower frequency of naïve CD4^+^and CD8^+^ T cells and naïve B cells than young ChRM before infection. *P* values were determined using the Mann-Whitney t-test. (**b**) The correlation matrix constructed by package corrplot with R reveals the relationships between the markers of immunosenescence and SIV disease progression during infection. (**c**) The correlation matrix reveals the relationships between characteristic values before and after infection. Pre, value before infection; Peak, peak value during infection; Nadir, minimum value during infection. The blue and red indicate positive and negative correlations, respectively (see color bar). The results of Spearman’s test (two-tailed, unadjusted) with a *P* value ≥ 0.05 were considered not significant (shown as cross).

As shown in Fig. 5b, strong associations were observed among most markers of immunosenescence and disease progression, especially between the naïve CD4^+^ T cell count and the CD4^+^ T cell count (r = 0.79) or CD4/CD8 ratio (r = 0.60, *P* < 0.0001 for all) during infection. In contrast, the frequency of Ki67^+^CD8^+^ naïve T cells showed a positive correlation with plasma viral load (r = 0.63, *P* < 0.0001).

Before infection, the old ChRM had a severe immunosenescence phenotype, as demonstrated by the significantly lower frequency of naïve CD4^+^ T cells (25.78%) and naïve CD8^+^ T cells (18.50%), and the insignificantly lower frequency of naïve B cells (29.54%) compared with that in young macaques (47.20%, 34.33% and 42.28%; *P* = 0.0047, 0.0127 and 0.0813, respectively) (Fig. 5a). Notably, these characteristics of pre-infection, peak and nadir levels had stronger correlations with one another than was found during the dynamic changes during infection. We observed that the frequency of naïve CD4^+^ T cells before infection had a strong inverse correlation with the peak level of exhaustion (PD-1^+^) CD4^+^ T cells (r = −0.74, *P* = 0.0023) and a strong positive correlation with the nadir number of CD4^+^ T cells (r = 0.86, *P* < 0.0001). While the frequency of naïve CD8^+^ T cells before infection had a strong inverse correlation with the peak level of PD-1^+^CD8^+^ T cells (r = −0.77, *P* = 0.0014). We also found that the peak expression of Ki67 in CD4^+^ or CD8^+^ naïve T cells was significantly associated with the nadir CD4^+^ T cell count (r = −0.76 and −0.73, *P* = 0.0017 and 0.0029, respectively). Interestingly, the frequency of Ki67^+^CD8^+^ naïve T cells showed a significant correlation with the set-point level of viral loads (r = 0.65, *P* = 0.0112) (Fig. 5c). These results indicated that the rapid SIV progression in ChRM may be largely due to their dramatic development of immunosenescence both pre- and post-infection.

### Old ChRM generate a more robust host response than do young macaques

According to the data from studies of pathogenic SIV infection and aging in rhesus macaques, we chose 46 innate immune genes that were likely to be differentially expressed between young and old SIV-infected macaques^17, 29^. These genes are involved in the interferon (IFN) and pattern recognition receptor (PRR) signaling pathways. We also measured the mRNA expression levels of chemokine and regulatory genes in PBMCs. Thirty genes that were significantly up-regulated with differential expression between the age groups were identified by ANOVA (Supplementary Fig. S4). A KEGG enrichment analysis demonstrated that these genes mainly participate in the antiviral response and interact closely with one another; they also partially represent the innate host response (Supplementary Fig. S5).

**Figure 6:**
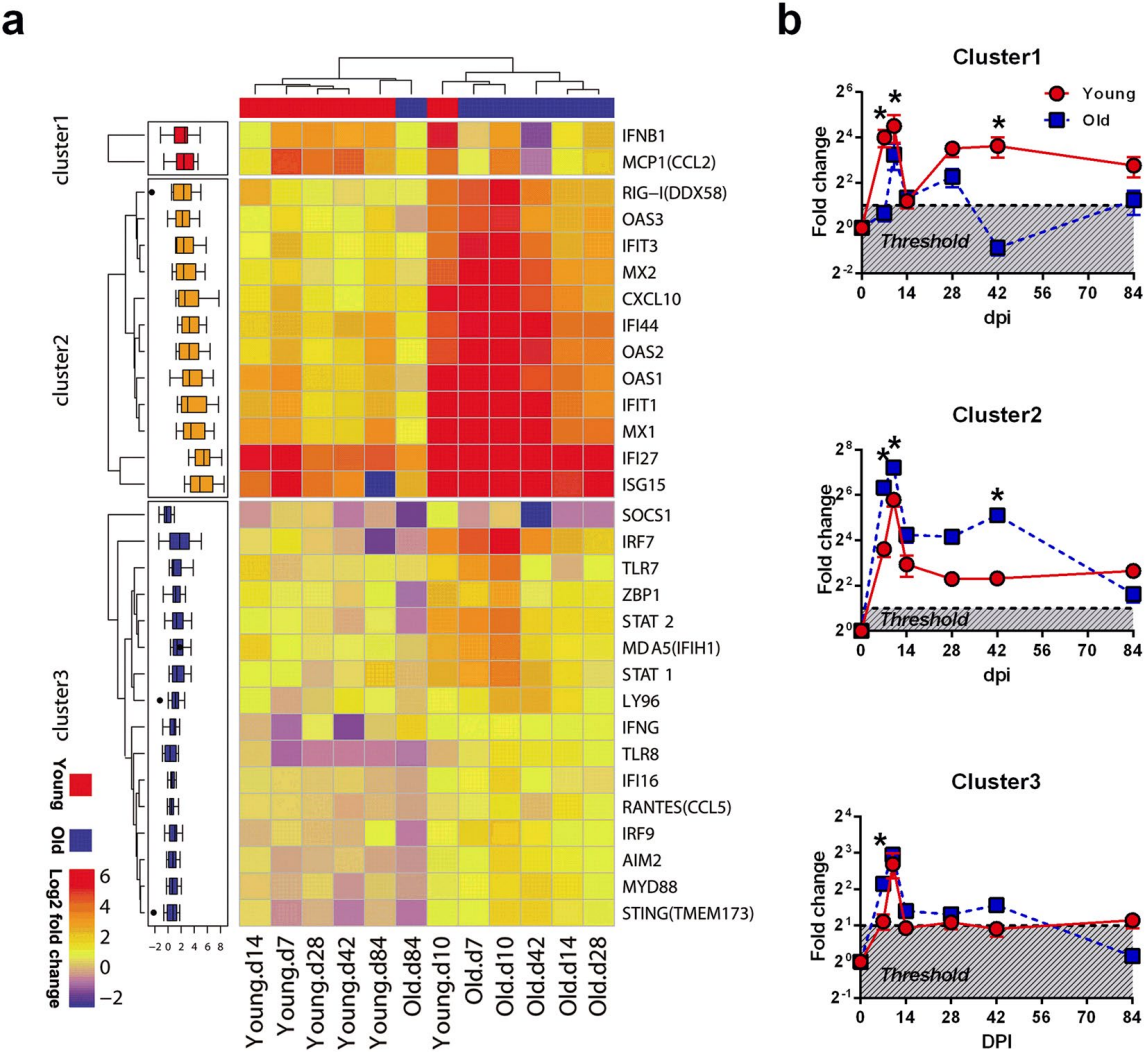
Acute SIV infection induces a robust innate host response in old ChRM. (**a**) Hierarchical clustering of 30 genes significantly induced by SIV infection (dpi = 0, 7, 10, 14, 28, 42 and 84) and differentially expressed between the young (n = 6) and old (n = 6) ChRM. Clustering was performed on the average of log2 ratios of mRNA expression relative to levels before infection by the RT-PCR-based ΔΔCt method. The genes were clustered on the basis of their expression value across samples using Euclidean distance and k-mean clustering. The progressive increases in mean log2-fold-change are represented by blue to red colors. (**b**) Graphs that display the characteristics of the three distinct clusters revealed in the left heat map. The threshold represents the credible induction by SIV infection for more than a 2-fold up-regulation of gene expression. The data are shown as the mean ± SEM. *P < 0.05 between the young (red circle) and old (blue square) groups, as determined by uncorrected Fisher’s LSD test post two-way ANOVA.

As shown in Fig. 6a, the hierarchical clustering of these 30 genes indicated that samples collected at 10 dpi exhibited the greatest expression changes after infection and that the old ChRM showed a rapid up-regulation of most genes at 7 dpi. Subsequently, the mRNA expression decreased to intermediate levels and was distinct in the different age groups at 14, 28 and 42 dpi. At 84 dpi, the expression of partial genes had already declined to baseline levels in both young and old ChRM. These genes were further organized into three clusters by K-means clustering. Genes within cluster 1 (*IFNB1* and *CCL2*) were rapidly up-regulated to a greater extent at 7 dpi in the young ChRM and decreased to less than twice the baseline in the old ChRM after 28 dpi. Within cluster 2, *RIGI*, a cytoplasmic sensor of viral RNA, and 10 interferon-simulated genes (ISG) were more highly induced in the old ChRM than in the young macaques during the acute phase. The expression characteristics of the genes within cluster 3 were more complex. *SOCS1*, the only differentially expressed gene among the 7 regulators in this study, was significantly down-regulated in the old ChRM compared with the young ChRM. *CCL5*, *IFI16* and *TLR7* were evaluated only in old ChRM. Other genes within cluster 3 were significantly induced to a level more than twice the baseline only at 10 dpi in the young macaques; this induction occurred much earlier (at 7 dpi) and more robustly in the old ChRM during the acute phase (Fig. 6b).

**Figure 7:**
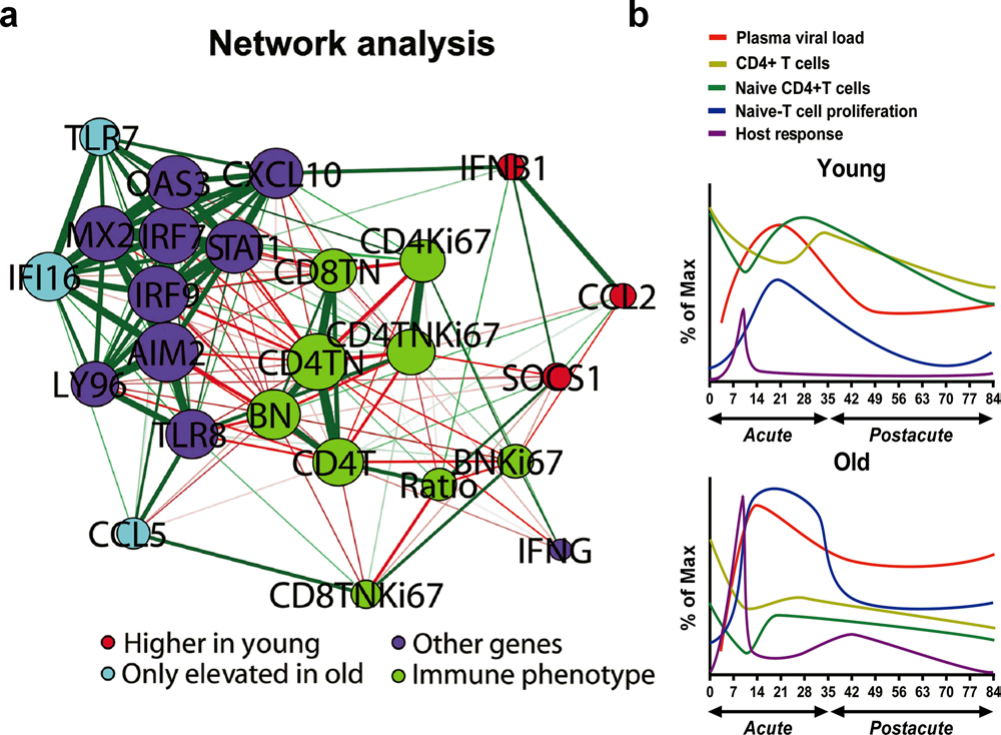
Systematic analysis of data of immunosenescence, disease progression and host response. (**a**) The correlation network of immunophenotype and gene expression data was constructed by using the qgraph package with R. The 4 groups of nodes are colored on the basis of data types. The groups include genes expressed higher in the young group (*IFNB1*, *CCL2* and *SOCS1*), genes elevated in only the old group (*TLR7*, *IFI16* and *CCL5*), other genes significantly correlated with immunophenotype (*OAS3*, *CXCL10*, *MX2*, *IRF7*, *STAT1*, *IRF9*, *AIM2*, *LY96*, *TLR8* and *IFNG*) and an immunophenotype that differed between the young and old groups (CD4T, CD4^+^ T cell number; Ratio, CD4/CD8; CD4TN, naïve CD4^+^ T cell number; CD8 TN, naïve CD8^+^ T cell number; CD4Ki67, % of Ki67^+^CD4^+^ T cells; CD4TNKi67, % of Ki67^+^CD4^+^ T cells; CD8TNKi67, % of Ki67^+^CD8^+^ T cells; BN, naïve B cell number; BNKi67, % of Ki67^+^ naïve B cells). The node size indicates the relative strength value according to a centrality analysis. The thicker lines indicate more correlated genes. The green lines represent significantly positive Pearson correlation coefficients ≥ 0.40, and the red lines represent significantly negative Pearson correlation coefficients ≤ −0.40. (**b**) Model of immunosenescence, disease progression and host response in ChRM that demonstrates their variation differences during early SIV infection as detailed in the text. Immunosenescence is shown by the numbers of naïve CD4^+^ T cells and the percentage of Ki67^+^ naïve T cells. The plasma viral loads and CD4^+^ T cell numbers represent the level of disease progression. The innate host response is defined by the fold-changes of differentially expressed genes. The smooth curves were constructed by a spline fit method using GraphPad Prism 6 software.

The naïve CD4^+^ T cell count, an important marker of immunosenescence that links the innate host response and disease progression accompanied by the proliferation of naïve lymphocytes, was at the center of the network constructed on the basis of the correlations between gene expression and immunophenotype during early SIV infection (Fig. 7a). The naïve lymphocyte count, especially the naïve CD4^+^ T cell count, rather than other immunophenotypes, had a significant strong correlation with the fold-changes of various genes. Notably, the expression of *SOCS1* displayed a strong correlation with the CD4/CD8 ratio (r = 0.47, *P* = 0.0003) and the proportion of Ki67^+^CD4^+^ naïve T cells (r = −0.42, *P* = 0.001), whereas positive correlations were observed between *CCL5* expression and the proportion of Ki67^+^CD8^+^ naïve T cells (r = 0.52, *P* < 0.0001). The CD4^+^ T cell count was inversely associated with the expression of *TLR8* (r = −0.45, *P* = 0.0005) and *LY96* (r = −0.43, *P* = 0.0008) (Supplementary Fig. S6).

## Discussion

SIVmac239-infected ChRM are characterized by a slow progression that is similar to that in HIV-1-infected humans. For example, in both adult humans and ChRM, the plasma viral load reaches its peak 2 weeks post-infection and then gradually declines to a low level throughout the chronic stage, CD4^+^ T cells progressively decline after acute infection, and the progression to AIDS in the first year of infection is very rare^22, 30^. In our study, the plasma viral loads in old ChRM were also at a moderate peak and low set-point level during early infection and rapidly rose to peak levels and remained high as the infection progressed. However, the dynamics of the CD4^+^ T cells in old ChRM was more similar to that of SIV-infected PTM, which are characterized by a fast progression with a rapid and complete of loss CD4^+^ T cells within 60 weeks after infection^31^. Furthermore, old ChRM had a low level of B cells, as is also seen in chronic HIV-1infection^6^. Because fewer CD4^+^ T cells and B cells and the inversion of CD4/CD8 are strong predictors of mortality and morbidity in old individuals^9^, it is not surprising that old ChRM showed a high risk of fast progression during early infection.

Interestingly, old ChRM had higher naïve T cell proliferation levels. Because the increased Ki67 expression might reflect the proliferation of cells entering an activated state, studies have suggested that elevated Ki67 expression in T cells is associated with an increased risk of AIDS in both HIV-1- and SIV-infected individuals^31–33^. Furthermore, a previous report has demonstrated that the expression of Ki67 is elevated in peripheral naïve T cells from old rhesus macaques, thus partially representing a dramatic increase in homeostatic turnover and a loss of the naïve T-cell pool^34^. Together with the decrease in naïve lymphocytes in old ChRM and the strong association between Ki67^+^ naïve T cells and disease progression, these observations suggest that the naïve lymphocytes in old ChRM have a rapid turnover and exhaustion, thereby probably contributing to disease progression.

Numerous studies have found that the loss of naïve CD4^+^ T cells occurs fairly early during HIV-1 infection and precedes the loss of immune homeostasis and AIDS progression. Thus, a naïve T-cell subset may play an important role in delaying HIV progression^35–37^. In our study, the low frequency of naïve T cells before infection in ChRM predicted a robust immune activation and rapid SIV progression. Moreover, in accordance with the robust turnover in the naïve subset, the rapid decrease in naïve CD4^+^ T cell and naïve B cell numbers that was induced by SIVmac239 infection in old ChRM may have contributed to the gradual absence of their immune defense. Similarly to what has been shown in elderly individuals, there is some evidence that direct HIV infection and HIV-induced immune activation destroy the microenvironments supporting immune renewal, reduce the thymic and myeloid output and finally, result in the disruption of normal immune homeostasis and the loss of naïve lymphocytes in the periphery^38–40^. Our data demonstrated that old ChRM exhibited an SIV-induced decrease of thymic and myeloid output after 2 months post-infection. In fact, in the chronic stage, SIV infection induced a deep impairment of immune homeostasis, thus resulting in a scarcity of naïve T cells in the peripheral blood of old ChRM (unpublished data).

The gene expression data from old ChRM displayed a rapid, strong and persistent interferon and pathogen recognition response to SIVmac239. Notably, distinct from nonpathogenic SIV infection with a transient elevated expression of ISGs in the acute phase, the expression of ISGs is persistently higher in HIV-infected individuals and SIVmac239-infected macaques^29, 41^. This phenomenon was also seen in the young and especially in the old ChRM. Other studies have also found that most ISGs are significantly elevated in old rhesus macaques infected with various viruses such as H1N1 and SARS-CoV. This elevation is due to the highly activated upstream NF-κB transcription factor, primarily because of increased viral replication and inflammation^18, 19^. Recent studies have demonstrated that a sufficient type I interferon (IFN-I) response during acute infection suppresses the depletion of CD4^+^ T cells, SIV transmission and active HSCs. However, the persistent activation of the IFN response after acute SIV infection may functionally impair HSCs with hematopoietic defects and decrease the protection from viruses caused by IFN desensitization^42, 43^. Interestingly, *SOCS1*, which encodes a member of cytokine-inducible negative regulators of cytokine signaling that act as modulators of IFN-γ action (ENTREZ gene summary), was slightly induced by SIVmac239 in ChRM but was quickly down-regulated in old macaques. Specifically, *IFNB1* expression was highly induced in young ChRM and may have partially prevented the depletion of CD4^+^ T cells. As a corollary to the above, and in line with the negative correlation between ISG expression and naïve lymphocyte number, it is possible that the IFN response plays a key role in the initiation of the anti-viral program and the homeostatic proliferation that compensates for the massive depletion of naïve lymphocytes. However, the uncontrolled and highly activated IFN response may finally induce anergy in the IFN signal and silence the antiviral activity in the hosts.

SIVmac239 infection in old ChRM also induced strong expression of several PRRs, including *TLR7*, *TLR8*, *RIG-I*, *MDA5*, *AIM2*, *IFI16*, *TMEM173*, *ZBP1* and *LY9*6, which function as extracellular signaling sensors for dsRNA, ssRNA, dsDNA and lipopolysaccharides (LPS) generated by viral and microbial translocation. Because they are associated with the up-regulation of *MYD88*, *IRF7*, *IRF9*, *STAT1* and *STAT2*, these genes can activate signaling cascades and lead to the activation of the NF-κB and IFN-I responses^44–48^. However, the highly significant defect in the Toll-like receptor (TLR)-induced cytokine production in monocytes and plasmacytoid dendritic cells (pDC) and in the pDC-induced functional activity of CD8^+^ T cells observed in the elderly may result from decreased TLR surface expression and dysfunctional IFN regulatory factor 7 (IRF7)^49–51^. Thus, integrating the contradictory findings, we suspect that immunosenescence contributes to the sensitivity to SIVmac239 and causes a high level of viral replication, which in turn elevates the host response and immune activation and ultimately accelerates disease progression in old ChRM. However, to make the best use of PBMC resources, we selected only the 6 old macaques with higher PBMC numbers and compared their gene expression levels with those in the 6 young animals. Therefore, our study may only partially reflect the relationships between gene expression and other parameters. Moreover, the T cell-specific chemokine RANTES, which plays an important role in sustaining CD8 T cell responses^52^, is markedly induced in old ChRM and associates with the homeostatic proliferation of T cells. Thus, it may be an effective marker for disease progression in old patients infected with HIV.

Together, the data first suggest that immunosenescence before infection, which is marked by a clear constriction of the naïve subset and the accumulation of memory subsets in lymphocytes, primarily accelerates disease progression in old ChRM, which is characterized by a rapid increase in the plasma viral load and a decrease in peripheral CD4^+^ T cells. Simultaneously, this process induces the robust proliferation of naïve T cells and the host response to compensate for the severe exhaustion of naïve CD4^+^ T cells (Fig. 7b). However, the persistent immune activation and lack of immune regulation in old ChRM eventually leads to increased immunosenescence and accelerated disease progression that first prompts these events. In conclusion, aging results in increased disease severity after SIV infection, even before the chronic phase. In addition, old ChRM may serve as an excellent model for elderly HIV-infected people and may thus promote research relating to the pathogenesis of HIV/AIDS and aimed at identifying therapeutic strategies for elderly patients.

## Methods

### Animals and sample collection

This study was carried out in strict accordance with the regulations of the American Association for Assessment and Accreditation of Laboratory Animal Care (AAALAC) at the Kunming Primate Research Center, Kunming Institute of Zoology, Chinese Academy of Sciences. All of the procedures were performed according to the guidelines approved by the Ethics Committee of the Kunming Institute of Zoology.

In this study, 6 young (mean age = 7.0 years old) and 12 old (mean age = 18.2 years old) male ChRM without BV, SRV and STLV infection were intravenously (i.v.) infected with 3000 TCID_50_ of SIVmac239 as previously described^53^. Four old macaques (96045, 96051, 96085 and 96649) that reached IACUC-defined endpoints were humanely euthanized with an overdose of barbiturates after their weights decreased by 30% because of persistent diarrhea (Supplementary Table S1). All of the animals were Mamu-A*01 negative, but 2 of the young (07067 and 08351) and 3 of the old (96061, 97077 and 99993) macaques still had at least one Mamu-B*03, Mamu-B*08 and Mamu-B*17 allele related to slow SIV progression. EDTA-stabilized venous blood samples were collected within 84 days post-infection. Plasma was collected, and peripheral blood mononuclear cells (PBMCs) were isolated by gradient centrifugation using Ficoll Density Centrifugation Media (GE Healthcare Life Sciences).

### Antibodies for flow cytometry

Human mAbs that cross-react with rhesus macaques were used at their optimal concentrations in this study. Anti-CD38-FITC (clone AT-1) was obtained from StemCell Technologies. Anti-PD-1-PE (eBioJ105) and anti-CD27-PE-Cy5 (O323) were obtained from eBioscience. Anti-CCR5-PE (3A9), anti-CD14-APC (M5E2), anti-CD28-APC (CD28.2), anti-CD20-APC-H7/FITC (2H7), anti-CD3-APC-Cy7 (SP34-2), anti-CD4-PerCP-Cy5.5 (L200), anti-CD8-PE-Cy7 (RPA-T8), anti-CD80-FITC (L307.4), anti-CD86-PE (FUN-1), anti-CD95-FITC/PE-Cy7 (DX2), anti-CXCR4-PE-Cy7 (12G5), anti-HLA-DR-APC (G46-6) and anti-Ki67-PE (B56) were all obtained from BD Biosciences. Polyclonal anti-IgD-biotin was obtained from Southern Biotech, and streptavidin-PE-Cy7 was obtained from BioLegend.

### Immunophenotyping

The absolute number of CD4^+^ T, CD8^+^ T and B cells in whole blood was measured using BD Trucount absolute counting tubes according to standard flow cytometric procedures, as described previously^23^. Leukocytes were surface^−^labeled with fluorescence^−^conjugated antibodies after treatment with red blood cell lysis buffer to delineate the naïve (CD95^dim^CD28^−^), central memory (CD95^high^CD28^+^) and effector memory (CD95^+^CD28^−^) CD4^+^ and CD8^+^ T^−^cell subsets, as well as the naïve (CD27^−^IgD^+^), un^−^switched memory (CD27^+^IgD^+^), switched memory (CD27^+^IgD^−^) and double negative (CD27^−^IgD^−^) B^−^cell subsets. To evaluate the immune activation of each subset, anti^−^CD38, anti^−^HLA^−^DR, anti^−^PD^−^1, anti^−^CD80 and anti^−^CD86 antibodies were also added for surface staining. Surface^−^labeled cells were fixed and permeabilized using a Cytofix/Cytoperm kit (BD Biosciences) for Ki67 staining to detect proliferation. The acquisition of at least 1,000,000 events was performed using a FACSVerse flow cytometer (BD Biosciences), and data analysis was performed using FlowJo software (Tree Star) (Supplementary Fig. S1).

### Viral load

The number of viral RNA copies in the plasma was directly determined by one-step quantitative RT-PCR (ViiA7 Real-Time PCR System, Applied Biosystems; RNA-direct Real Time PCR Master Mix, TOYOBO) using pure viral RNA, as previously described^22^.

### Quantitative RT–PCR

RNA and DNA were extracted from thawed cryopreserved PBMCs by using RNAisoPlus (TAKARA). mRNA levels were measured with the SYBR Green q-PCR method as previously described^54^. Briefly, purified RNA was reverse-transcribed into cDNA with a PrimeScript RT Reagent Kit (TAKARA) and then amplified with SYBR Premix Ex Taq II (TAKARA) in a ViiA7 Real-Time PCR System. Primers specific for RPL13A mRNA were used as an endogenous standard to normalize the samples. Fold-change was calculated by dividing the normalized post-infected sample quantity by the normalized pre-infected control quantity according to the ΔΔCt method.

Genomic DNA (250–500 ng) was used for the absolute quantification of the T-cell receptor rearrangement excision circle (TREC), the kappa-deletion recombination excision circle (KREC) and the TCR α constant gene (TCRAC). The quantification was carried out with Premix Ex Taq (Probe qPCR) (TAKARA) according to the manufacturer’s instructions. The concentration of TREC or KREC in the PBMCs was calculated with the following formula: TRECs or KRECs per PBMC = mean quantity of TREC or KREC/(mean quantity of TCRAC/2). The number of TRECs/T cells or KRECs/B cells can be calculated according to the flow cytometry-based frequency of T or B cells in PBMCs. The specific primer and probe sequences for each mRNA, DNA and SIVgag RNA are given in Supplementary Table S4.

### Statistical analysis

ANOVA was used to define the parameters that were significantly affected by SIV infection or that differed between the young and old macaques. Comparisons between day 0 and each day post-infection were performed with paired t-tests. The correlations between two variables in the correlation matrix were evaluated with Spearman’s test. The analyses were performed using R 3.3.2, SPSS 22 (IBM) and GraphPad Prism 6 (GraphPad software). The data in the text are shown as average values. The threshold for statistical significance was a two-tailed *P* value of 0.05.

## Electronic supplementary material

Supplementary Information
