# Supplementary material for: Accelerated disease progression and robust innate host response in aged SIVmac239-infected Chinese rhesus macaques is associated with enhanced immunosenescence
Source: Sci Rep. 2017 Feb 24;7:37. doi: 10.1038/s41598-017-00084-0 (PMC5428349; doi:10.1038/s41598-017-00084-0)
Supplement: Supplementary file 1 — Supplementary Information [file 41598_2017_84_MOESM1_ESM.pdf]

Accelerated disease progression and robust innate host response in aged SIVmac239-infected Chinese rhesus macaques is associated with enhanced immunosenescence

Hong-Yi Zheng[1,2], Ming-Xu Zhang[1,3], Min Chen[1,3], Jin Jiang[1,3], Jia-Hao Song[1,4], Xiao-Dong Lian[1,3], Ren-Rong Tian[1], Xiao-Liang Zhang[1], Lin-Tao Zhang[1], Wei Pang[1], Gao-Hong Zhang[1], Yong-Tang Zheng[1,2,3,5,*]

1. Key Laboratory of Animal Models and Human Disease Mechanisms of Chinese Academy of Sciences &Yunnan Province, Kunming Institute of Zoology, Chinese Academy of Sciences, Kunming, Yunnan 650223, China.
2. School of Life Sciences, University of Science and Technology of China, Hefei, Anhui 230026, China.
3. Kunming College of Life Science, University of Chinese Academy of Sciences, Kunming, Yunnan 650204, China.
4. Institute of Health Sciences, Anhui University, Hefei, Anhui 230601, China.
5.Kunming Primate Research Center, Kunming Institute of Zoology, Chinese Academy of Sciences, Kunming, Yunnan 650223, China.

***Corresponding author**: Prof. Yong-Tang Zheng, Kunming Institute of Zoology, Chinese Academy of Sciences, Kunming, Yunnan 650223, China, Tel and Fax: +86-871-65195684, E-mail: zhengyt@mail.kiz.ac.cn.

Supplementary Table S1. The basic information of Chinese rhesus macaques (ChRM) in this study.

Group	Animal	Age	DPI with peak viral load	Status after 84 days post infection	Time of death (DPI)	Cause of death	Weights before infection (Kg)	Weights when died (Kg)
Young	07067	8	14	alive			8.5	
	08035	7	21	alive			8.1	
	08051	7	14	alive			7.2	
	08309	7	14	alive			8.5	
	08351	7	21	alive			9.65	
	09435	6	21	alive			6.2	
Old	95095	20	14	alive			9.35	
	96045	19	10	dead	49	persistent diarrhea	7.14	4.8
	96051	19	14	dead	84	persistent diarrhea	7.3	5.18
	96057	19	14	alive			7.45	
	96061	19	10	alive			9.41	
	96085	19	14	dead	42	persistent diarrhea	12.46	8.8
	96649	19	14	dead	84	persistent diarrhea	11.2	7.1
	97077	18	14	alive			10.65	
	98325	17	14	alive			10.5	
	98379	17	14	alive			7.78	
	99377	16	14	alive			7.68	
	99993	16	14	alive			8.57	

DPI, days post infection.

Supplementary Table S2. The average number of T cell subsets and frequency of activated, proliferative and CCR5+ cells in each T cell subset from peripheral blood in young (n = 6) and old (n = 8) ChRM during SIVmac239 infection. The markers significantly affected by SIV infection (DPI) and differed between young and old groups (Age) are identified by two-way ANOVA. *P* value of < 0.05 was considered significant.

	Young macaques (mean)												
**DPI**	**d0**	**d3**	**d7**	**d10**	**d14**	**d21**	**d28**	**d35**	**d42**	**d49**	**d56**	**d70**	**d84**
**CD4TN(No.)**	504.10	447.10	492.73	329.75	510.88	393.26	443.05	531.46	406.29	474.75	309.49	339.78	233.80
**CD4CM(No.)**	513.38	421.77	492.65	419.16	433.24	249.49	334.53	335.10	285.96	278.15	215.34	270.30	201.01
**CD4EM(No.)**	51.09	134.13	111.51	49.92	56.55	48.97	54.53	45.03	18.65	45.90	18.33	31.68	33.53
**CD8TN(No.)**	227.72	255.15	315.72	176.57	276.41	274.86	267.67	336.31	251.55	297.37	185.59	224.53	170.41
**CD8CM(No.)**	158.72	211.95	199.53	160.25	228.71	202.14	261.01	279.65	249.96	252.55	163.69	171.07	166.64
**CD8EM(No.)**	276.48	502.31	454.47	260.32	299.02	355.39	453.22	363.18	223.20	375.19	183.47	215.68	219.14
**CD38+HLA-DR+CD4**	1.80	1.48	3.45	1.28	1.04	1.63	1.66	2.39	2.57	2.68	2.94	3.21	2.45
**PD-1+CD4**	26.45	30.75	30.48	31.33	26.60	26.32	29.27	26.97	24.83	28.08	27.88	30.93	33.30
**CD38+HLA-DR+CD8**	6.39	8.37	14.08	8.92	8.05	16.23	16.08	15.85	16.35	13.51	17.87	14.52	12.73
**PD-1+CD8**	39.45	43.07	43.27	41.88	39.32	48.13	50.05	48.50	46.08	49.40	47.37	45.03	48.72
**Ki67+CD4**	2.10	7.74	7.94	10.60	4.56	6.97	11.74	8.55	6.59	6.09	9.58	8.45	13.43
**Ki67+CD4TN**	0.70	1.58	1.97	2.08	1.44	2.33	1.96	1.81	1.89	1.26	1.98	1.09	1.72
**Ki67+CD4CM**	3.79	14.61	14.70	17.55	8.72	14.24	26.92	20.73	13.10	13.33	20.10	16.07	27.52
**Ki67+CD4EM**	2.41	9.78	7.66	15.05	7.95	12.21	9.18	7.63	5.17	4.96	12.55	9.97	17.92
**Ki67+CD8**	3.31	10.49	9.64	18.94	12.85	21.08	24.18	20.62	9.47	11.71	15.53	12.78	16.93
**Ki67+CD8TN**	1.03	2.43	1.87	2.85	3.38	10.10	7.86	8.25	2.52	2.02	3.01	1.38	1.93
**Ki67+CD8CM**	6.87	22.60	18.17	24.68	24.33	35.92	36.93	32.02	17.20	21.97	26.17	22.02	28.63
**Ki67+CD8EM**	3.85	11.48	12.86	26.91	18.31	22.53	27.47	23.72	8.45	12.89	17.98	15.67	22.22
**CCR5+memory CD4**	10.09	4.807	7.008	5.67	7.281	5.501	7.509	6.989	6.707	5.584	6.04	7.249	8.528

	Old macaques (mean)													Two way ANOVA		
**DPI**	**d0**	**d3**	**d7**	**d10**	**d14**	**d21**	**d28**	**d35**	**d42**	**d49**	**d56**	**d70**	**d84**	**Age**	**DPI**	**Age*DPI**
**CD4TN(No.)**	214.00	207.37	145.85	86.17	185.05	163.15	177.91	192.11	173.36	225.70	158.23	174.88	126.96	**1.83E-25**	**4.89E-03**	**ns**
**CD4CM(No.)**	601.15	502.52	396.05	295.16	320.95	218.14	258.64	282.33	252.19	314.33	251.52	291.46	248.44	**ns**	**1.57E-08**	**ns**
**CD4EM(No.)**	54.70	90.68	97.12	69.73	69.64	53.31	63.56	50.94	36.60	36.25	24.58	61.21	49.39	**ns**	**6.86E-06**	**ns**
**CD8TN(No.)**	145.00	169.39	152.05	94.25	239.51	179.28	219.96	224.81	187.48	200.28	152.39	139.00	109.84	**1.51E-05**	**ns**	**ns**
**CD8CM(No.)**	200.38	224.60	183.80	214.92	289.08	217.58	278.94	289.97	306.36	283.98	247.99	255.88	260.69	**1.93E-02**	**ns**	**ns**
**CD8EM(No.)**	475.02	496.83	565.71	729.20	520.51	727.74	761.13	614.22	410.19	557.54	453.97	532.73	482.48	**4.95E-06**	**ns**	**ns**
**CD38+HLA-DR+CD4**	2.31	1.79	3.69	3.04	1.92	2.24	3.01	4.58	3.60	4.37	4.98	5.29	4.78	**1.23E-05**	**3.25E-04**	**ns**
**PD-1+CD4**	34.89	35.30	35.89	38.59	29.30	31.05	32.36	35.08	33.25	33.84	32.74	34.83	37.84	**1.37E-05**	**ns**	**ns**
**CD38+HLA-DR+CD8**	7.65	6.59	12.47	13.57	11.18	18.16	18.36	16.81	17.67	17.16	21.13	19.61	18.49	**ns**	**2.52E-03**	**ns**
**PD-1+CD8**	57.34	55.76	57.21	63.94	62.89	66.45	64.54	64.83	61.70	64.35	59.93	64.59	68.67	**7.77E-14**	**ns**	**ns**
**Ki67+CD4**	5.65	8.28	10.13	10.20	4.71	10.95	14.27	15.61	11.90	12.79	11.97	12.77	16.83	**1.48E-05**	**6.85E-08**	**ns**
**Ki67+CD4TN**	1.61	2.25	2.87	1.55	1.83	3.81	3.32	5.12	3.57	3.70	3.14	3.34	2.76	**4.53E-07**	**4.01E-02**	**ns**
**Ki67+CD4CM**	7.67	12.19	12.68	12.48	6.87	17.54	21.56	23.35	16.93	16.66	18.17	17.39	19.93	**ns**	**1.78E-12**	**ns**
**Ki67+CD4EM**	5.87	8.51	8.91	7.55	16.32	16.26	15.84	14.94	14.01	16.00	14.50	13.09	14.70	**ns**	**ns**	**ns**
**Ki67+CD8**	6.59	7.23	11.52	11.03	19.93	21.61	27.18	19.69	12.62	14.21	15.32	16.27	21.66	**ns**	**4.14E-09**	**ns**
**Ki67+CD8TN**	1.80	2.14	4.49	4.08	15.69	16.12	15.94	15.01	6.05	5.77	5.58	5.99	6.94	**1.74E-05**	**2.49E-07**	**ns**
**Ki67+CD8CM**	10.56	13.18	16.40	17.99	27.11	35.51	42.48	33.79	20.29	23.24	26.54	28.01	34.26	**ns**	**1.47E-15**	**ns**
**Ki67+CD8EM**	6.97	7.15	11.44	9.49	17.77	18.43	24.89	16.17	9.83	11.51	12.52	14.05	18.74	**ns**	**2.76E-08**	**ns**
**CCR5+memory CD4**	18.60	12.44	12.00	11.41	8.26	8.95	9.69	8.77	6.71	10.02	9.10	9.29	14.51	**< 0.0001**	**0.0057**	**ns**

TN, naïve T cells (CD95-CD28+); TCM, central memory T cells (CD95+CD28+); TEM, effector memory T cells (CD95+CD28-).

Supplementary Table S3. The average number of B cell subsets and levels of activation and proliferation markers in each B cell subset from peripheral blood in young (n = 6) and old (n = 8) ChRM during SIVmac239 infection. The markers significantly affected by SIV infection (DPI) and differed between young and old groups (Age) are identified by two-way ANOVA. *P* value of < 0.05 was considered significant.

	**Young macaques (mean)**														
**DPI**	**d0**	**d3**	**d7**	**d10**	**d14**	**d21**	**d28**	**d35**	**d42**	**d49**	**d56**	**d70**	**d84**			
**BN(No.)**	593.72	660.51	391.89	141.09	290.72	281.36	250.51	304.86	304.95	326.38	298.80	279.34	232.58			
**BUM(No.)**	358.73	194.78	175.92	99.80	122.55	194.67	285.82	229.15	263.47	260.30	219.22	169.93	143.21			
**BSM(No.)**	316.82	222.74	229.32	145.59	166.84	227.27	301.82	309.84	374.43	412.91	284.56	337.66	338.92			
**BDN(No.)**	73.16	149.54	58.25	14.22	26.48	37.68	16.06	25.18	25.36	30.99	17.53	28.66	16.20			
**Ki67+B**	8.41	7.81	6.66	8.67	5.95	9.84	11.99	14.51	11.16	16.37	15.44	17.85	23.65			
**Ki67+BN**	5.02	5.28	4.59	5.91	4.24	4.28	7.29	8.34	5.45	5.70	5.48	5.59	7.13			
**Ki67+BUM**	13.34	15.12	11.25	18.28	8.52	18.98	17.63	18.47	14.04	19.05	18.16	19.40	22.28			
**Ki67+BSM**	10.49	8.50	5.78	6.20	3.52	11.04	12.48	18.05	15.40	24.75	22.42	24.90	35.83			
**Ki67+BDN**	9.89	11.83	8.61	11.41	10.34	11.76	18.50	20.68	19.05	19.70	18.82	21.90	30.00			
**CD95+B**	48.01	47.45	44.87	60.85	49.98	51.90	52.38	51.58	59.65	55.67	58.37	59.70	63.70			
**CD80+B(MFI)**	416.42	408.67	397.67	463.00	389.83	418.67	466.67	501.33	556.17	549.83	558.33	536.50	582.25			
**CD86+B(MFI)**	691.49	726.83	662.33	761.00	777.00	1142.67	1282.00	1041.50	1251.17	1076.33	1145.83	905.67	1089.00			
	**Old macaques (mean)**													**Two way ANOVA**		
**DPI**	**d0**	**d3**	**d7**	**d10**	**d14**	**d21**	**d28**	**d35**	**d42**	**d49**	**d56**	**d70**	**d84**	**Age**	**DPI**	**Age*DPI**
**BN(No.)**	242.06	283.07	210.93	195.89	102.10	205.08	190.93	263.42	179.14	238.58	186.56	211.64	198.30	**3.95E-05**	**1.81E-03**	**ns**
**BUM(No.)**	230.53	194.73	177.30	205.98	106.06	189.67	281.22	244.91	255.93	194.56	223.83	188.84	208.04	**ns**	**ns**	**ns**
**BSM(No.)**	321.15	319.72	237.93	374.60	194.03	266.73	362.75	363.45	448.03	510.09	377.16	467.76	443.54	**ns**	**ns**	**ns**
**BDN(No.)**	50.79	80.90	31.57	28.15	15.86	23.30	19.26	87.26	57.22	68.46	30.24	117.29	48.13	**ns**	**ns**	**ns**
**Ki67+B**	8.91	8.78	11.40	9.49	8.03	17.86	17.36	16.54	11.95	14.89	15.80	17.48	24.13	**ns**	**2.90E-06**	**ns**
**Ki67+BN**	5.05	5.70	7.55	7.19	7.42	7.90	9.29	10.76	5.61	6.39	8.12	8.69	11.12	**5.75E-03**	**ns**	**ns**
**Ki67+BUM**	13.73	14.27	17.42	13.06	11.29	27.09	24.39	19.84	14.78	17.95	19.71	21.96	28.26	**ns**	**ns**	**ns**
**Ki67+BSM**	10.46	10.20	10.83	8.94	6.86	21.49	18.04	21.17	13.80	18.40	19.47	21.62	31.30	**ns**	**1.44E-10**	**ns**
**Ki67+BDN**	9.11	12.38	12.78	13.19	14.07	25.70	25.44	24.62	19.02	23.78	20.19	21.59	26.41	**ns**	**1.86E-05**	**ns**
**CD95+B**	50.37	46.78	47.40	60.98	55.02	50.62	50.90	48.55	59.87	53.55	54.57	62.35	63.12	**ns**	**4.97E-04**	**ns**
**CD80+B(MFI)**	440.41	435.75	426.13	479.20	414.75	448.00	505.63	517.38	540.38	572.75	559.75	578.71	543.43	**ns**	**1.74E-09**	**ns**
**CD86+B(MFI)**	729.01	682.38	676.38	699.40	988.50	1134.50	1307.75	1144.38	1486.50	1156.75	1319.63	1152.14	1095.57	**ns**	**8.81E-12**	**ns**

BN, naïve B cells (CD27-IgD+); BUM, un-switched memory (CD27+IgD+) B cells; BSM, switched memory B cells (CD27+IgD-); BDN, double negative B cells (CD27-IgD-).

Supplementary Table S4. Oligonucleotide primer sequences used for real-time PCR.

Gene	Forward (5' - 3')	Reverse (5' - 3')
**RPL13A**	AAGGTGTTTGACGGCATCCC	CTTCTCCTCCAAGGTGGCTGT
**IFNA2**	CCTGGCACAAATGAGGAGAAT	GGAACTGGTTGCCAAACTCC
**IFNB1**	GAGGAAATTAAGCAGCCGCA	AGTCTCATTCCAGCCAGTGC
**IFNK**	ATTGCTGGCACCCTATCCCT	TTCTTGGGGCAACTCAAAAGC
**IFNW1**	TTCAGGTTCCCCCAGGAGAT	ATGGTCTAGGAGGGTCGTGT
**IFNG**	GAGTGTGGAGACCATCAAGGA	ACTGCTTTGCGTTGGACATT
**IRF7**	AGCTGCATGTTCCTGTACG	TCAGCAGTTCCTCCGTGTAG
**IRF9**	TGAGCCACAGGAAGGTACAG	ACGCCCGTTGTAGATGAAGG
**STAT1**	TCATCAGCAAGGAGCGAGAG	CGCATGGAAGTAAGGTTCGC
**STAT2**	GGCTCTCAGTTGGCAGTTCT	CGCTTAGTGAAGTCAGCCCA
**ADAR**	ATGACCAGCCCGAAGGTATG	AGCTCGCCAATCTTCCTGAC
**SOCS1**	AACTCGCACCTCCTACCTCT	AAATAAAGCCAGAGACCCTCCC
**SOCS3**	CCCCAGAAGAGCCTACTACA	ACAGAGATGCTGAAGAGTGGC
**CXCL10**	TGCTGCCTTGTCTTTCTGACT	ATGCTGATGCAGGTACAGCG
**IFI27**	ACTCTCCGGGTTGACCAGAT	TGGCACGGTTCTCTTCTCTG
**IFI44**	TGATAAACGCTGGTGTGGTACA	TGGACTTCCTCTAGCTTGGAC
**IFIT1**	AGGAAACACCCACTTCGGTC	CTGCCCTTTTGTAGCCTCCT
**IFIT3**	GAAGCCGAAGGAGAGCAGTT	CCAATGCCCGTTGAAACAGT
**ISG15**	AAAGATCGCCCAGAAGATCGG	TCATCGCACTTGTCCACCAC
**MX1**	AAAGCCCAGAATACCATCGCC	TGTCAGGAGGTTGATTGCCC
**MX2**	AACTTGGTGGTGGTTCCCTG	GGTGCCCCTGTCCATTAGAT
**OAS1**	GCAGAAAGAGGGCGAGTTCT	GTGCTTGACTAGGCGGATGA
**OAS2**	CCTGGAGCTGGTCACACAAT	CCTGGTTTTCTGCAACTGGC
**OAS3**	TTGAGGCATGTCAACGGGAG	CAGCACGTCAAAGTCCACAC
**TLR3**	CATTGGGCCTCTTCCTGAAC	CCGAACGCTTGTGTTTGCTA
**TLR7**	TGGAAATTGCCCTCGTTGTT	AGCGCATCAAAAGCATTTACAG
**TLR8**	GGGCTGCAAAATCTCACTAAAATAA	CATGCCAGGATTTCCGTTCT
**TLR9**	GAAGACTTCAGGCCCAACT	CACGGTCACCAGGTTGTT
**DDX58**	TGGAAGCATTCAAAGTCAATGG	GCAATGTCAATGCCTTCATCA
**IFIH1**	ACAAAAACACGGCAGAGTGC	GATGGTGGGCTTTGACTCCT
**MAVS**	GCCTTCTGTGTTCACCAATCC	CATGCTAGCAGGCACTTTGGA
**IFI16**	GTGGCAACTGAGAATGAAGTCTTC	TTGAGATGGCAATGATCTTCTTTG
**MYD88**	CCAGCATCCTGAGGTTCATCA	CAAGGCGAGTCCAGAACCAA
**TMEM173**	ACAGCATCTATGAGCTTCTGGAGAA	ACATGGCAAACAAAGTCTGCAA
**LY96**	ACTCTTCCAAAGCGCAAAGA	ATTCACAGTCTCTCCCTTCAGAG
**RBCK1**	GATGCTCAGATGCACACTGTCA	TGGGAAGCCATAGTCCAGAAA
**SARM1**	TGCAGGCTGTGCTTACTTTCA	AAGCGGATGATCTTCTCAATGG
**NLRX1**	TGTTGCCATCCAGATGTGTTG	GGATGAGGAGAAAGCACCAATG
**TOLLIP**	GCGAGGAGGACCTGAAAGC	CAGCACCGAGCGGATCAC
**CXCL9**	CCACATCCCACTCACAACAG	GCTGAGCAAACATCCTGTCA
**CCL2**	CCCCAGTCACCTGCTGTTAT	CAAAACATCCCAGGGGTAGA
**CCL3**	TCGAGCCCACATTCCGTCAC	GCTTTGGTGCCATGACTGCC
**CCL4**	CCAAACCAAAAGAAGCAAGC	AGAAACAGTGACAGTGGACC
**CCL5**	TCATTGCTACTGCCCTCTGC	CGTCGTGGTCAGAATCTGGG
**TREC**	TGC CAC ATC CCT TTC AAC CAT GCT	AGG TGC CTG TGC ATC ACC G
**KREC**	CAG CGC CCG TTA CGT TTC TG	GGA CTC CAG GAG CCA GCT CTT

**TCRAC**	AAT GAG ATC ATG TCC TAA CCC TGA TCC	ATT TAG AGC CTC TCA GCT GGT ACA C
**SIVgag**	TCG GTC TTA GCT CCA TTA GTG CC	GCT TCC TCA GTG TGT TTC ACT TTC
**Mamu-DRB**	GCCTCGAGTGTCCCCCCAGCACGTTTC	GCCGCAGCTTTCACCTCGCCGCTG
**Mamu-A*01**	GACAGCGACGCCGCGAGCCAA	GCTGCAGCGTCTCCTTCCCC
**Mamu-B*03**	TTCGTGCGGTTCGACAGT	GTTCCATCTCCTCCTGGCCTA
**Mamu-B*08**	CGTGAGGCGGAGCAGGTC	CCACAGCTCCGATGAACACAG
**Mamu-B*17**	AGAGCAGCGGAGAGCCTACCT	GCTGCACATGGCACGTGTATC
	**Probes(5' - 3')**	
**TREC**	FAM-ACG CCT CTG GTT TTT GTA AAG GTG CTC ACT-TRAMA	
**KREC**	FAM TCT GCA CGG GCA GCA GGT TGG-TAMRA	
**TCRAC**	FAM-TCC CAC AGA TAT CCA GAA CCC TGA CCC-TRAMA	
**SIVgag**	FAM-CTT CTG CGT GAA TGC ACC AGA TGA CGC-TAMRA	

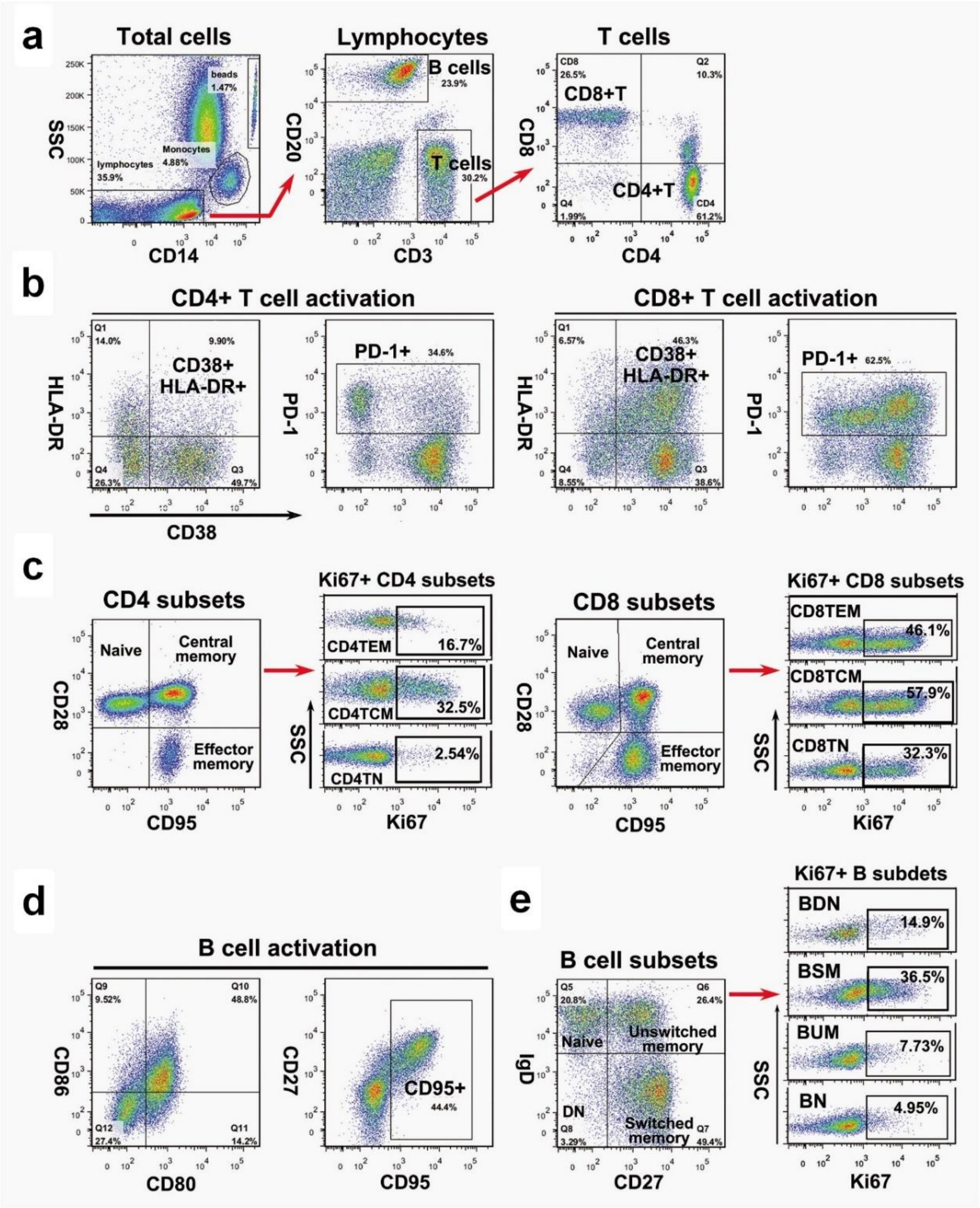

Supplementary Figure S1. Gating strategy for flow cytometry analysis. (**a**) In the cell counting analysis, cells were first gated for lymphocytes (CD14^{-}SSC^{low}), monocytes (CD14^{+}SSC^{med}) and trucount beads (bright fluorescent and high SSC signal). The lymphocyte gate is further analyzed for their expression of CD3 and CD20 to determine T cell (CD3^{+}CD20^{-}) and B cell (CD3^{-}CD20^{+}). CD4^{+} T cells (CD4^{+}CD8^{-}) or CD8^{+} T cells (CD4^{-}CD8^{+}) is then determined from this gated population. The calculation formula of absolute cell number is as follows: cells/μL = (total beads count × gated cell count) / (gated beads count × blood volume). (**b**) Gating strategy for identifying activated CD4^{+} and CD8^{+} T cells (CD38^{+}HLA-DR^{+} or PD-1^{+}). (**c**) Gating strategy for the naïve (CD95^{dim}CD28^{+}), central memory (CD95^{high}CD28^{+}) and effector memory (CD95^{+}CD28^{-}) subsets of CD4^{+} and CD8^{+} T cells and the proliferation level (Ki67 expression) of each this subset in peripheral blood. TN, naive subset; TCM, central memory subset; TEM, effector memory subset. (**d**) Gating strategy for identifying activated B cells (CD80, CD86 and CD95 expression). (**e**) Gating strategy for the naïve (CD27^{-}IgD^{+}), un-switched memory (CD27^{+}IgD^{+}), switched memory (CD27^{+}IgD^{-}) and double negative (CD27^{-}IgD^{-}) subsets of B cells and the proliferation level (Ki67 expression) of each this subset in peripheral blood. BN, naïve subset; BUM, un-switched memory subset; BSM, switched memory subset; BDN, double negative subset.

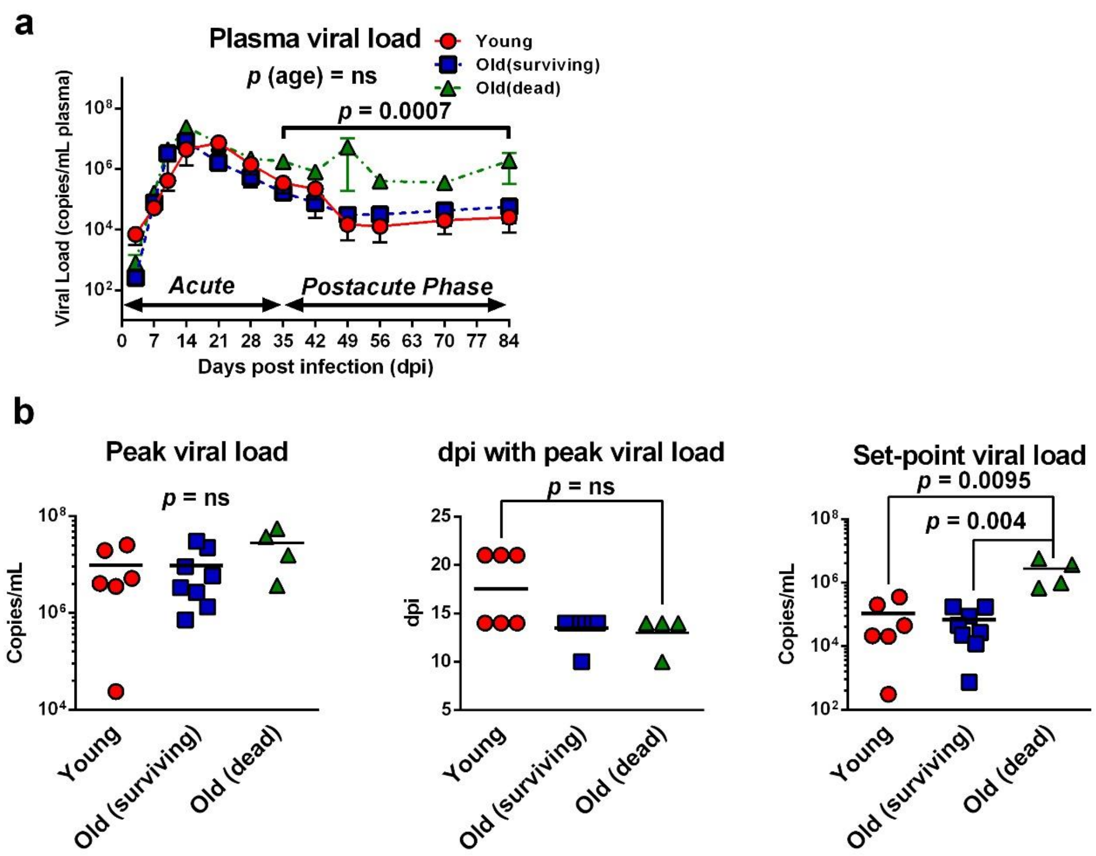

Supplementary Figure S2. Comparisons for viral loads among young, surviving old and dead old macaques during early SIV infection. (**a**) Plasma viral load is expressed as SIV RNA copies/ml and shown for three groups (6 young macaques, 8 surviving old macaques and 4 deceased old macaques). Data are shown as the mean ± SEM. Comparison among groups was done by two-way ANOVA. (**b**) Peak and set point levels of viral load and days with peak viral load were compared between the three groups. *P* values were determined using the Mann-Whitney t-test post Kruskal-Wallis test.

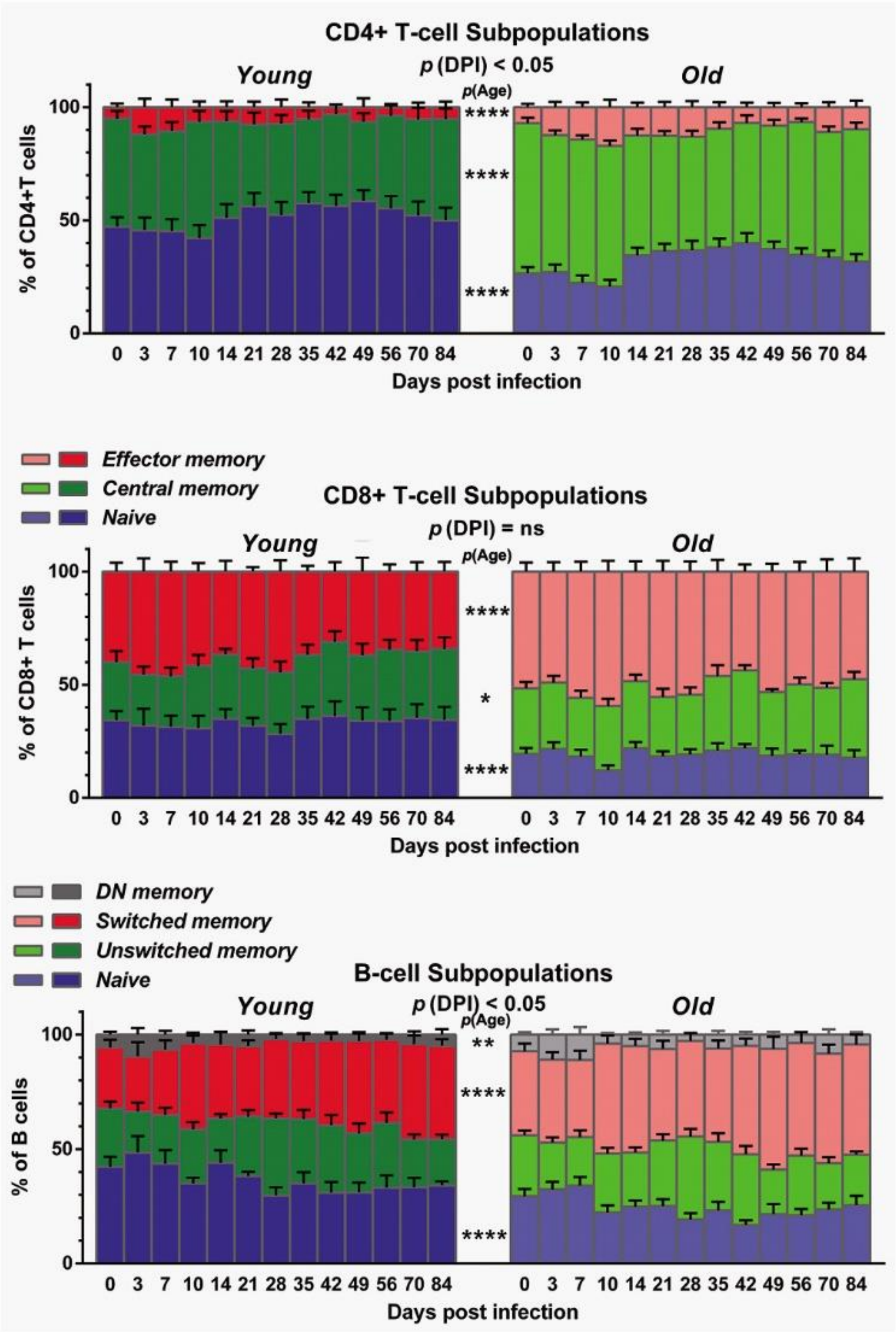

Supplementary Figure S3. Dynamics of T-cell and B-cell subsets during early SIV infection. Stacked error bars display the frequency of each $CD4^+$ T-cell, $CD8^+$ T-cell and B-cell subset in peripheral blood from young (n = 6) and old macaques (n = 12) during 84 days post infection (DPI). T cell subsets were defined as: naïve (deep blue for young group and lake blue for old group), central memory (deep green for young group and grass green for old group), and effector memory cells (bright red for young group and pink for old group). B cell subsets were defined as: naïve (deep blue for young group and lake blue for old group), un-switched memory (deep green for young group and grass green for old group), switched memory (bright red for young group and pink for old group) and double negative memory cells (dark grey for young group and grey for old group). Data are shown as mean with SEM. p(DPI) < 0.05, statistically significant differences over time of every subsets by two-way ANOVA. * $p < 0.05$, ** $p < 0.01$, *** $p < 0.001$ and **** $p < 0.0001$, one corresponding subset has statistically significant differences between young and old by two-way ANOVA.

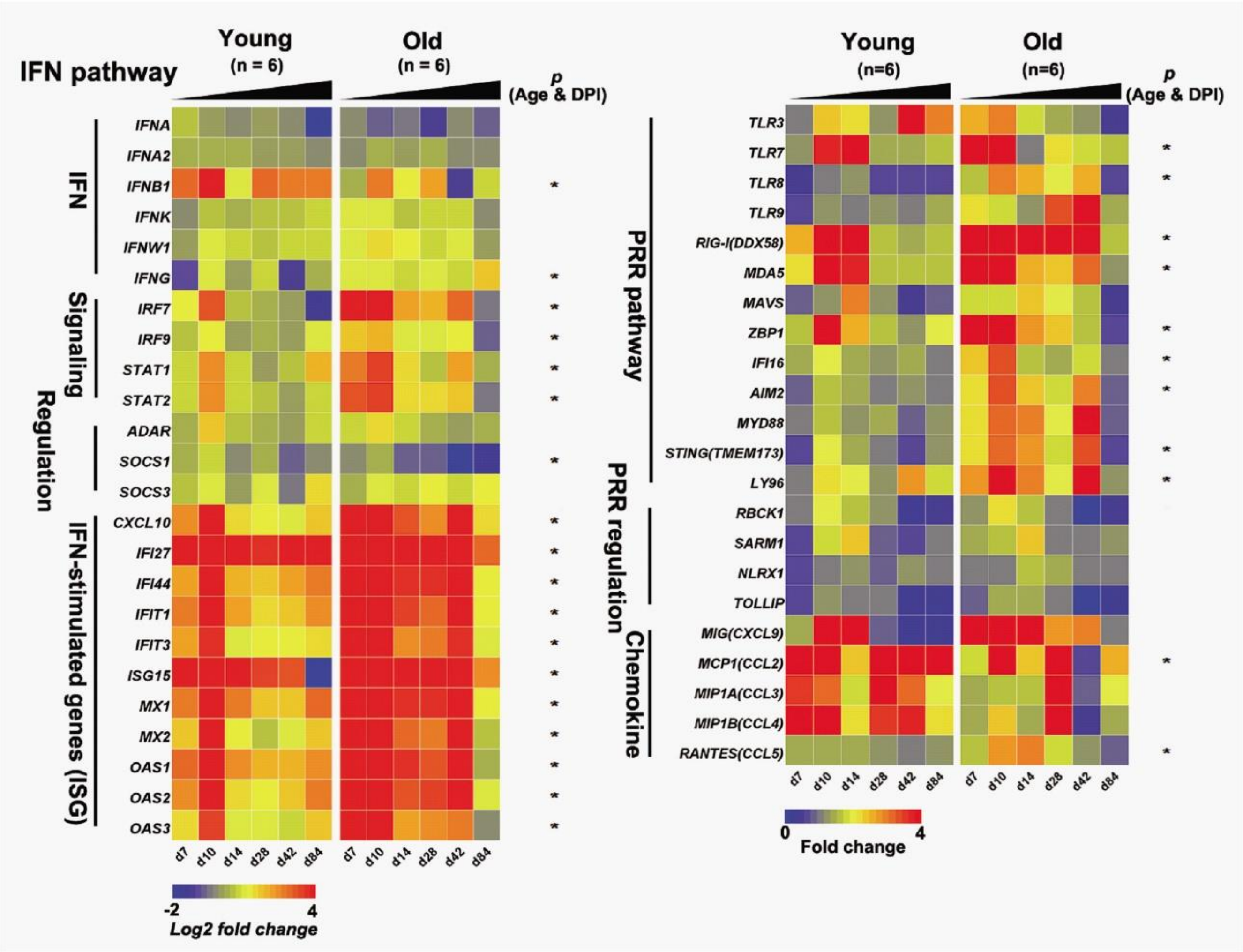

Supplementary Figure S4. Differentially expressed genes in PBMC from young versus old ChRM during SIV infection. Heat map showing the log2 or linger mean fold-change in mRNA expression of 46 genes involved in interferon signaling pathway, pattern recognition receptor signaling pathway, chemokines and regulation from 6 young macaques and 6 old macaques. 30 genes are up-regulated and differentially expressed within age groups during SIV infection. Relative mRNA expression was relatively quantified using RPL13A gene expression as reference and the ΔΔCt method by RT-PCR. Progressive increases in mean fold-change are represented by blue to red colors.*$p < 0.05$ between young and old group as well as over time by two-way ANOVA.

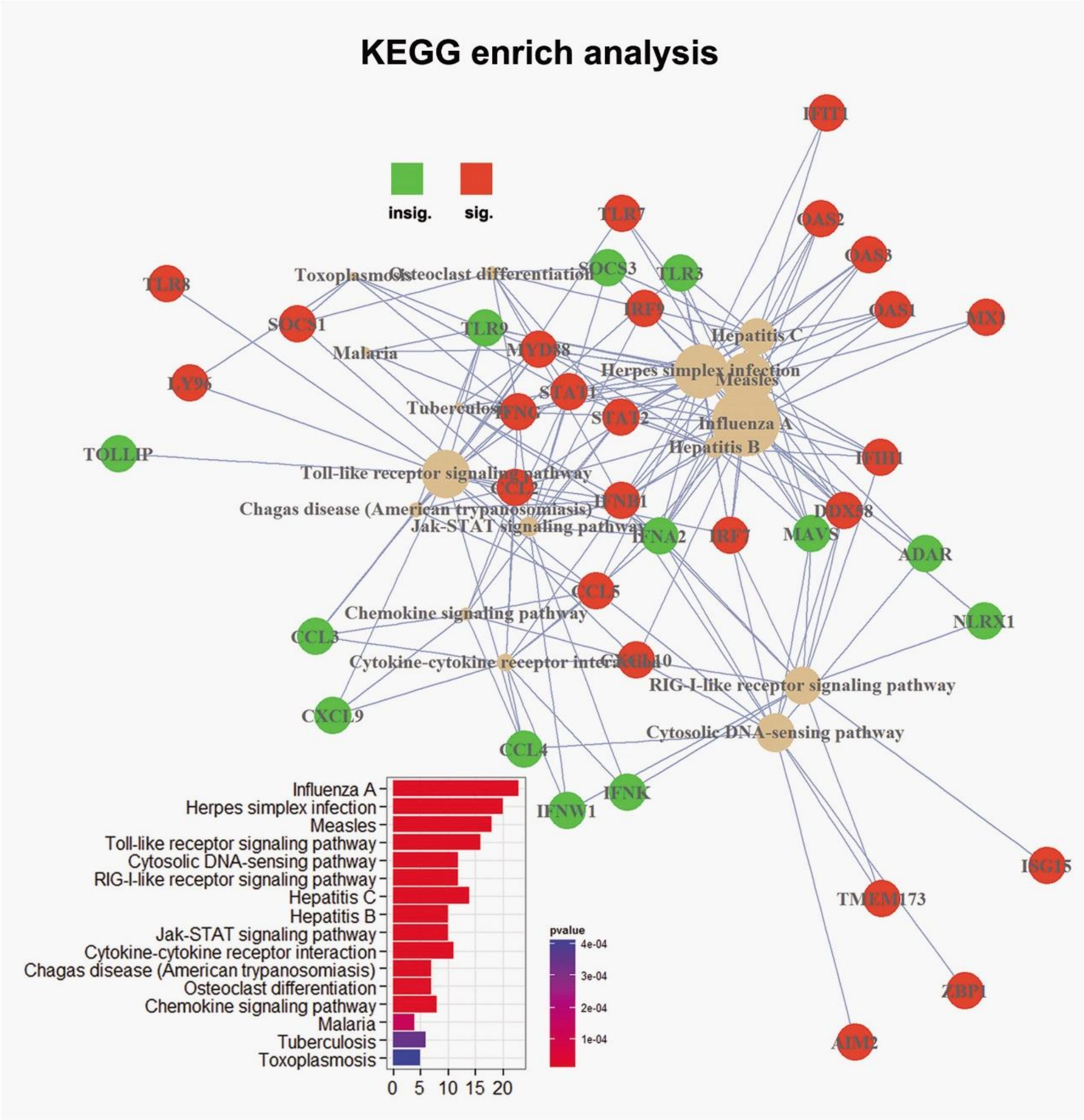

Supplementary Figure S5. Kyoto encyclopedia of genes and genomes (KEGG) pathway analysis within differentially expressed genes. Significantly enriched KEGG pathways for observed genes are shown as network and bar plot. The pathways having enrichment ($p< 0.01$) are presented. For each KEGG pathway, the bar shows the number of genes affected in each pathway in our dataset. Differentially expressed and other genes are indicated by red and green, respectively.

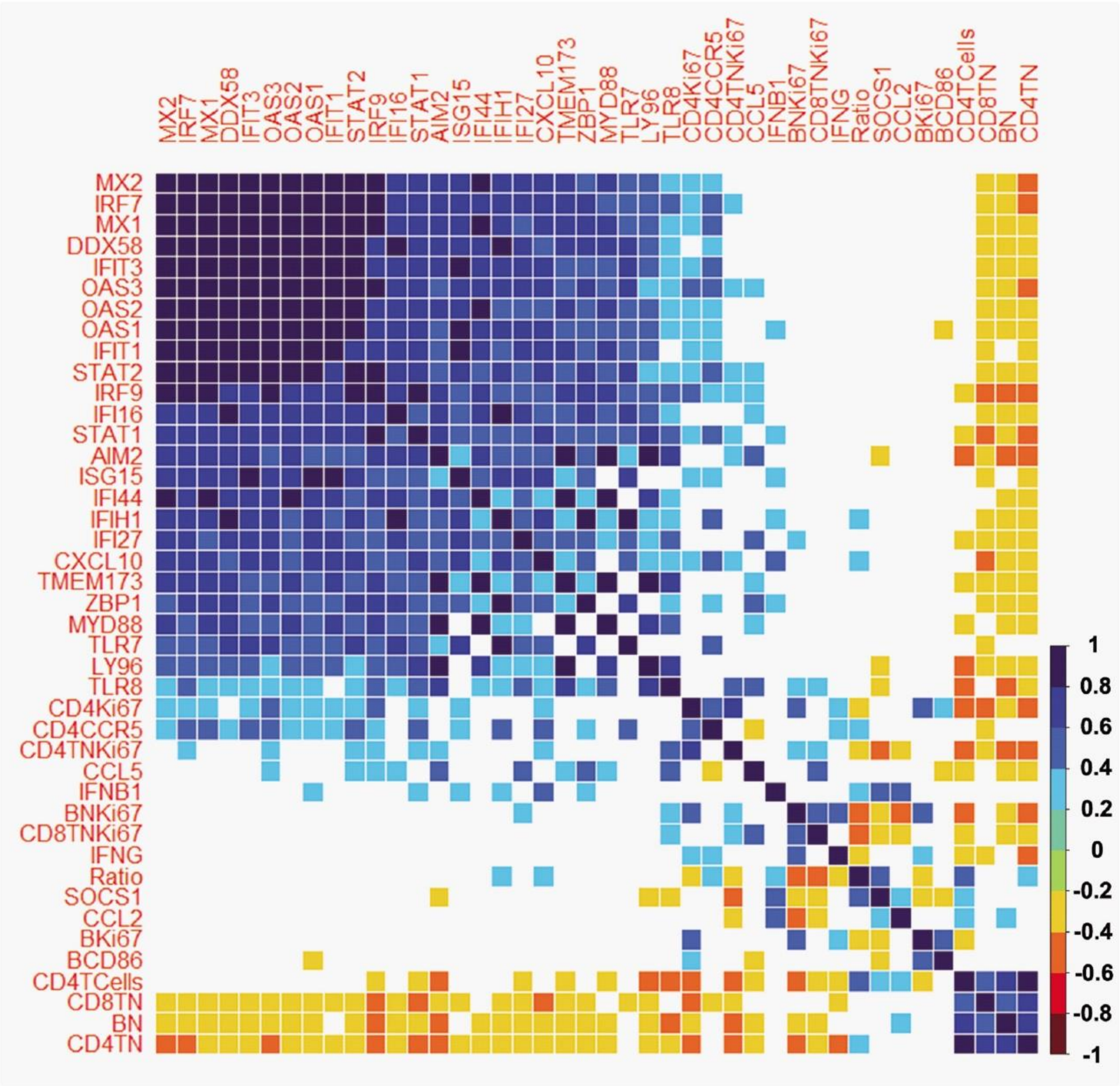

Supplementary Figure S6. Correlation matrix reveals relationships between immunological values and gene expression profiles. Blue and red indicate positive and negative correlations, respectively (see color bar).The results for the Pearson's correlation (two tailed, unadjusted), with a *P* value of $\geqslant$0.05 were considered not significant (shown in blank). CD4TCells, $CD4^+$ T cell number; CD4TN, naïve $CD4^+$ T cell number; CD8TN, naïve $CD8^+$T cell number; Ratio, CD4/CD8; BN, naïve B cell number; Ki67+CD4TN, % of $Ki67^+CD4^+$ naïve T cells; $Ki67^+$CD8TN, % of $Ki67^+CD8^+$ naïve T cells; $Ki67^+$CD4T, % of $Ki67^+CD4^+$ T cells; CD4CCR5, % of $CCR5^+CD4^+$ T cells; BKi67, % of $Ki67^+$ B cells; BCD86, . % of $CD86^+$ B cells.
